# Synergistic effects of halo-plaque forming phage cocktails against polymicrobial *Acinetobacter baumannii* and *Staphylococcus aureus*

**DOI:** 10.1128/spectrum.04009-25

**Published:** 2026-04-30

**Authors:** Ampapan Naknaen, Arrita Rueangrit, Wanna Sudhikaran, Arnon Chukamnerd, Sanicha Chumtong, Sarunyou Chusri, Rattanaruji Pomwised, Komwit Surachat, Kamonnut Singkhamanan, Ruttayaporn Ngasaman

**Affiliations:** 1 Department of Biomedical Sciences and Biomedical Engineering, Faculty of Medicine, Prince of Songkla University, Songkhla, Thailand; 2 Department of Pathology, Faculty of Medicine, Prince of Songkla University, Songkhla, Thailand; 3 Division of Infectious Diseases, Department of Internal Medicine, Faculty of Medicine, Prince of Songkla University, Songkhla, Thailand; 4 Division of Biological Science, Faculty of Science, Prince of Songkla University, Hat Yai, Songkhla, Thailand; 5 Translational Medicine Research Center, Faculty of Medicine, Prince of Songkla University, Songkhla, Thailand; 6 Faculty of Veterinary Science, Prince of Songkla University, Songkhla, Thailand; Universidad Tecnica de Ambato, Ambato, Ecuador

**Keywords:** halo-phage, synergistic effects, co-infection, *Acinetobacter baumannii*, *Staphylococcus aureus*

## Abstract

Antimicrobial resistance is a growing global health crisis, with polymicrobial infections caused by *Acinetobacter baumannii* and *Staphylococcus aureus* posing a significant healthcare challenge due to their synergistic virulence, multidrug resistance, high incidence, and association with severe outcomes. Accordingly, urgent alternative treatments are needed, with bacteriophage or phage therapy showing considerable promise. Here, we isolated six phage types based on plaque morphology, including small clear plaques (SCP; phiAR002 and phiAR004), medium clear plaques (MCP; phiAR010 and phiAR011), and hazy-halo plaques (HHP; phiAR014 and phiAR015). These phages demonstrate the ability to lyse a broad spectrum of several carbapenem-resistant *A. baumannii* strains collected from secondary and tertiary care hospitals in Thailand, highlighting their suitability for therapeutic applications. To broaden host range and enhance bactericidal activity, phage cocktails comprising the halo-forming phage (phiAR014) in combination with either SCP (phiAR002) or MCP (phiAR010), which are genetically and mechanistically distinct, were formulated and demonstrated synergistic effects, suppressing bacterial growth for up to 36 h. These relatively small myophages, SCP and MCP, possess larger genomes (around 165–188 kbp) compared with HHP (around 106 kbp), exhibit a longer latent period (30 min versus 20 min), and produce a greater number of progenies, yielding 678–882 PFU per infected cell compared with 255 PFU per infected cell for HHP. To demonstrate their therapeutic potential, the phage cocktails were further assessed in co-cultures of *A. baumannii* and *S. aureus* under both planktonic and biofilm conditions. Cocktails combining HHP with SCP or MCP exhibited significant synergistic antibacterial activity at MOIs of 10 and 100, with effects evident within 10 h and sustained for 24 h. Relative to individual phages and uninfected controls, the cocktails achieved greater bacterial reduction, decreasing *A. baumannii* by approximately 2 log₁₀ CFU/mL compared with single phages (and ~5 log₁₀ versus controls) and *S. aureus* by ~2 log₁₀ CFU/mL. Moreover, the cocktails significantly enhanced biofilm inhibition and eradication compared to single-phage treatments, demonstrating synergistic interactions. These findings indicate that halo phages represent promising therapeutic candidates with potent antibacterial and anti-biofilm activity against polymicrobial infections.

**IMPORTANCE:**

Phages are promising therapeutic agents due to their host specificity and ability to penetrate biofilms via structural and enzymatic mechanisms. The halo-plaque phenotype, indicative of depolymerase activity, facilitates the degradation of extracellular polysaccharides and cell wall components, thereby enhancing infectivity and biofilm penetration. Based on phenotypic and genomic characterization, halo-forming phage cocktails were developed and demonstrated effective suppression of polymicrobial growth and disruption of established biofilms in *A. baumannii* and *S. aureus* co-culture models. These cocktails exhibited synergistic inhibition of planktonic growth, biofilm formation, and biofilm eradication. Phage replication and lysis of *A. baumannii* may promote the release of putative depolymerases and/or cell wall hydrolases that could potentially contribute to matrix degradation; however, further investigation is required to elucidate the precise mechanisms and enzymatic functions involved. Together, these findings would pave the way for advancing halo-forming phages and support the targeted engineering of tail fiber or spike proteins for therapeutic applications in multidrug-resistant polymicrobial infections.

## INTRODUCTION

Polymicrobial infections, involving *A. baumannii* and *S. aureus*, are commonly encountered in clinical settings and are frequently associated with increased disease severity and adverse patient outcomes (1, 2). *A. baumannii* is classified by the World Health Organization (WHO) as a Priority 1 pathogen because of its intrinsic antibiotic resistance and rapid acquisition of additional resistance mechanisms (3). The synergistic interaction between *A. baumannii* and *S. aureus* can alter key fitness determinants by providing alternative carbon sources, compensating for lysine degradation and DNA repair pathways, and increasing reliance on ABC transporter gene (1, 2). Another major factor contributing to antibiotic treatment failure in various clinical settings is the challenge of managing biofilm formation in polymicrobial infections, which is linked to 65–80% of clinically significant bacterial infections (4, 5). The biofilm matrix acts as a protective barrier against host immune responses and antimicrobial agents, limiting antibiotic penetration and reducing bacterial susceptibility owing to slowed growth and altered physiology (6, 7). Given that the WHO has classified the development of new treatments for these pathogens as an urgent priority (3), the use of phages has emerged as a promising alternative strategy for combating multidrug-resistant bacterial infections (8–10).

Phages are viruses that specifically infect and replicate within bacteria, and their associated enzymes, such as endolysins and depolymerases, are being reconsidered for therapeutic use because of their high specificity, synergistic potential with antibiotics, unique mechanisms, safety, natural availability, and potential for genetic modification (11). To date, most *A. baumannii* phages have been found to target the bacterial capsule as their primary receptor, with few alternative receptors identified (8). This capsule plays a critical role in virulence by safeguarding the bacterium against host immune responses and conferring protection from antimicrobial agents, including antibiotics (12–14). Phage-derived depolymerase activity and tail-associated proteins can degrade the bacterial capsule polysaccharide layer and enhance the susceptibility to phages, certain antibiotics, and serum killing (15). In *A. baumannii* phages, enzymatic activity, often evidenced by halo formation surrounding the phage plaques, is commonly associated with tail fiber proteins (15, 16). These enzymes act as hydrolases and lyases that facilitate the initial stages of the phage infection cycle, including adsorption and DNA injection (17, 18). Consequently, phages exhibiting enzymatic activity can effectively eradicate biofilm-associated cells while simultaneously degrading the biofilm extracellular polymeric matrix (18, 19). This dual action not only enhances bacterial susceptibility to phage therapy but also improves the management of bacterial infections and enables the targeted removal of biofilms from medical devices, thereby broadening the therapeutic potential of phage applications (15, 20).

It is well-established that phages infecting *A. baumannii* exhibit a notably narrow host range (21, 22), typically targeting only a limited number of strains, often owing to the presence of lineages distinct from those circulating in other geographic regions (23). This limitation restricts their therapeutic applications. Therefore, phage cocktails have been employed to broaden the host range and alleviate the rapid emergence of bacterial resistance (24–26). In this study, we report the isolation of three phages—phiAR002, phiAR010, and phiAR014—that represent the main plaque morphologies: halo and non-halo types. These phages were used to investigate the following questions: (i) whether a phage cocktail composed of genetically diverse phages with distinct replication mechanisms can enhance lytic activity and effectively suppress bacterial growth, and (ii) whether a phage cocktail containing phages with halo-plaque morphology can effectively inhibit both planktonic cells and biofilm formation in co-cultures of *A. baumannii* and *S. aureus*. We hypothesized that phages with diverse genetic backgrounds and replication mechanisms would exhibit distinct infection kinetics, particularly with respect to the timing of lysis. Furthermore, we examined whether phage cocktails composed of halo-forming phages could improve lytic efficacy and biofilm disruption compared with individual phages. Our results demonstrate that a strategic combination of halo phages based on their putative depolymerase or cell wall hydrolase activity could reduce the bacterial revival and effectively combat polymicrobial infections, offering valuable insights for the rational design of therapeutically effective phage cocktails.

## RESULTS

### Phage isolation and infectivity profiles are correlated with plaque morphology and the genetic relatedness of the tested carbapenem-resistant *A. baumannii* (CRAB) strains

**Fig 1:**
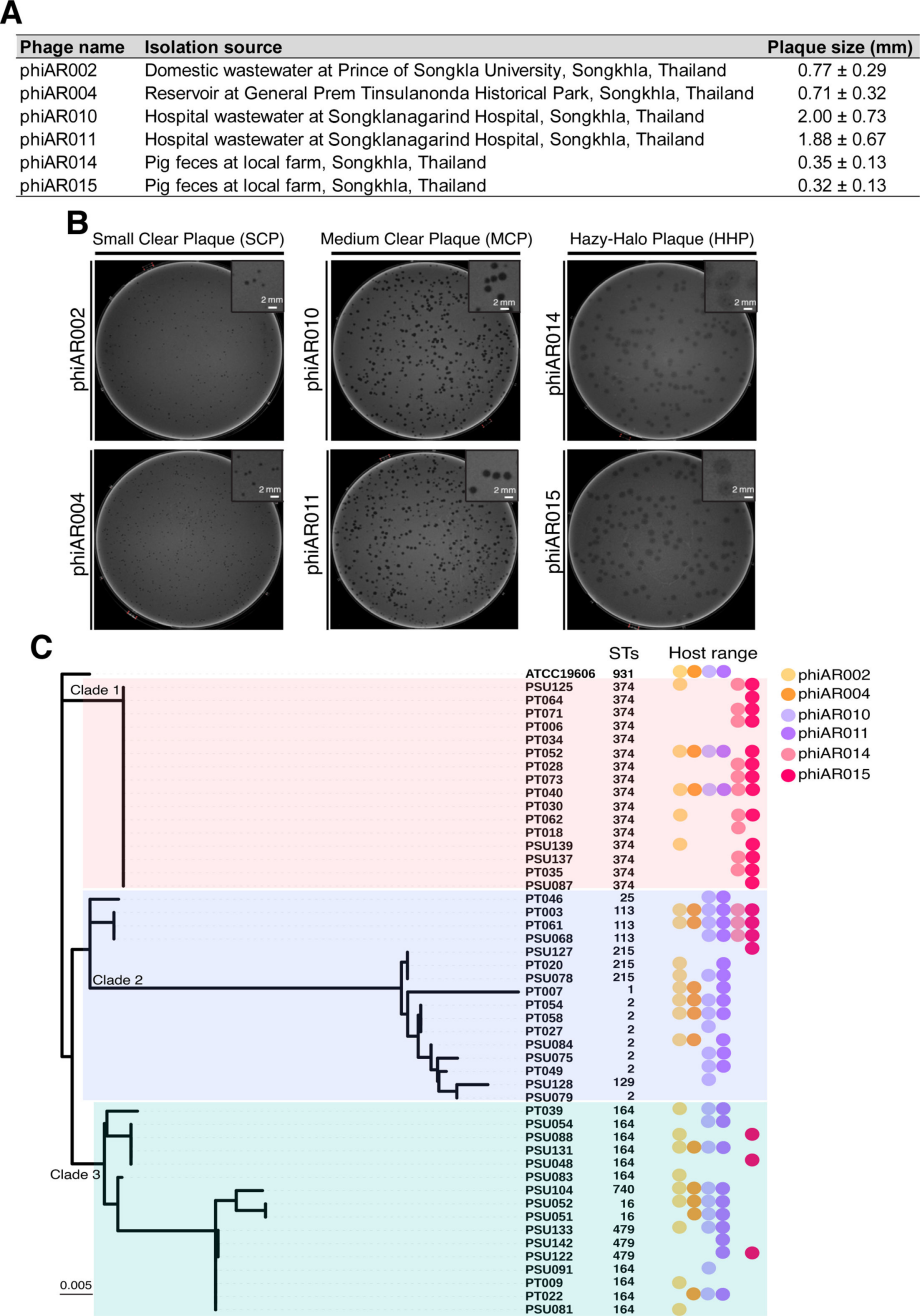
Phages were isolated from different sources and characterized based on plaque morphology and lytic activity against clinical carbapenem-resistant *A. baumannii* (CRAB). (**A**) The phage collection includes the phage name, the isolation source, and its plaque size. (**B**) Phage plaque morphology was observed on TSB supplemented with 0.35% agar and incubated at 37°C for 20 h. Phages were grouped based on plaque morphology, including small clear plaque (SCP) phiAR002 and phiAR004, medium clear plaques (MCP) phiAR010 and phiAR011, and hazy-halo plaques (HHP) phiAR014 and phiAR015. Magnified views of representative plaques are shown in the upper right corner of the image. Scale bars represent 2 mm. (**C**) Host range analysis revealed varying lytic spectra across CRAB strains, which were further correlated with phylogenetic relationships and sequence types (STs) determined by multilocus sequence typing (MLST) relying on the Oxford scheme. A phylogenetic tree was constructed using maximum likelihood with 1,000 bootstraps, followed by visualization via iTOL. The lytic profiles of phage show on the right based on plaque morphological types including the pinpoint plaques; phiAR002 (light orange circle) and phiAR004 (darker orange circle), the clear medium plaques; phiAR010 (light purple circle) and phiAR011 (darker purple circle) and the hazy-halo pinpoint plaques; phiAR014 (light pink circle) and phiAR015 (darker pink circle). The experiment was performed in three independent replicates.

Six phages were isolated using a parental host CRAB strain PT061 from wastewater samples and pig feces in Thailand (Fig. 1A). Based on plaque characteristics, phages can be categorized into three groups: small clear plaque (SCP) size (~0.71–0.77 mm), named phiAR002 and phiAR004; medium clear plaque (MCP) size (~1.88–2.00 mm), named phiAR010 and phiAR011; and hazy-halo pinpoint plaques (HHP) (~0.32–0.35 mm), named phiAR014 and phiAR015 (Fig. 1A and B). To further evaluate their potential for medical applications, we assessed the host range spectrum of 83 CRAB clinical strains isolated from both Songklanagarind and Phatthalung Hospitals (27), as well as strains of *Escherichia coli*, *Staphylococcus aureus*, *Pseudomonas aeruginosa*, and *Klebsiella pneumoniae*. Most CRAB isolates carried blaOXA-23, with some harboring blaNDM-1 or blaIMP-14, alongside multiple resistance genes, particularly against aminoglycosides, fosfomycin, and tetracyclines (27). All phages demonstrated the ability to infect several CRAB clinical isolates obtained from both hospitals, ranging at approximately 27–52% (13–25 out of 48) (Fig. 1C; Fig. S1A and B), whereas only the SCP phages were capable of infecting both *A. baumannii* DMST 2071 and ATCC 19606 (Fig. S1B). The MCP phages (phiAR010 and phiAR011) and the SCP phage (phiAR002) were able to infect a comparable range of CRAB clinical isolates from both hospitals (~42–55%). In contrast, phage phiAR004 (SCP), phiAR014 (HHP), and phiAR015 (HHP) showed greater infectivity toward CRAB isolates from Phatthalung Hospital (~36–50%) compared to those from Songklanagarind Hospital (~13–38%).

To examine whether the phage host range was associated with the genetic relatedness of CRAB, we integrated a phylogenetic tree and sequence types (STs), as determined by multilocus sequence typing (MLST), with the host range spectrum (Fig. 1C). The results showed that ~81% (13 out of 16) of CRAB isolates from Songklanagarind Hospital belonged to clade 3 and were primarily classified as sequence type (ST) 164, whereas clade 1 was predominantly composed of ST 374 CRAB isolates from Phatthalung Hospital, representing ~75% (12 out of 16). Clade 2 exhibited a relatively even distribution of CRAB isolates from both hospitals, with the predominant sequence types being ST 2, ST 113, and ST 215. Interestingly, HHP phages (phiAR014 and phiAR015) were able to infect the majority of clade 1 CRAB strains, ~87% (14 out of 16), whereas both non-halo phages (SCP and MCP) predominantly infected clade 3 strains, accounting for ~69% (11 out of 16). Furthermore, MCP phages (phiAR010 and phiAR011) were capable of infecting ~81% (13 out of 16) of clade 2 CRAB strains. These CRAB strains were further compared with publicly available genomes (*n* = 151) from the European Nucleotide Archive (ENA) database, which represents a wide range of geographical and ecological sources based on MLST. The analysis revealed that the CRAB strains from Thailand were closely related to those from Vietnam, Singapore, Australia, India, and Nigeria (Fig. S1C). This finding gives rise to the possibility that these phages may also demonstrate effectiveness against CRAB strains from these regions; however, further investigation will be necessary to confirm this potential. Our results indicate that the patterns of phage infectivity were associated with both plaque morphology and genetic relatedness of the host strains, highlighting the diversity and specificity of phage-host interactions within this clinical isolate collection. These findings support the suitability of these phages for therapeutic applications.

### Phage killing ability and phage cocktails combining HHP phages with SCP or MCP phages effectively suppressed bacterial growth and delayed bacterial revival

**Fig 2:**
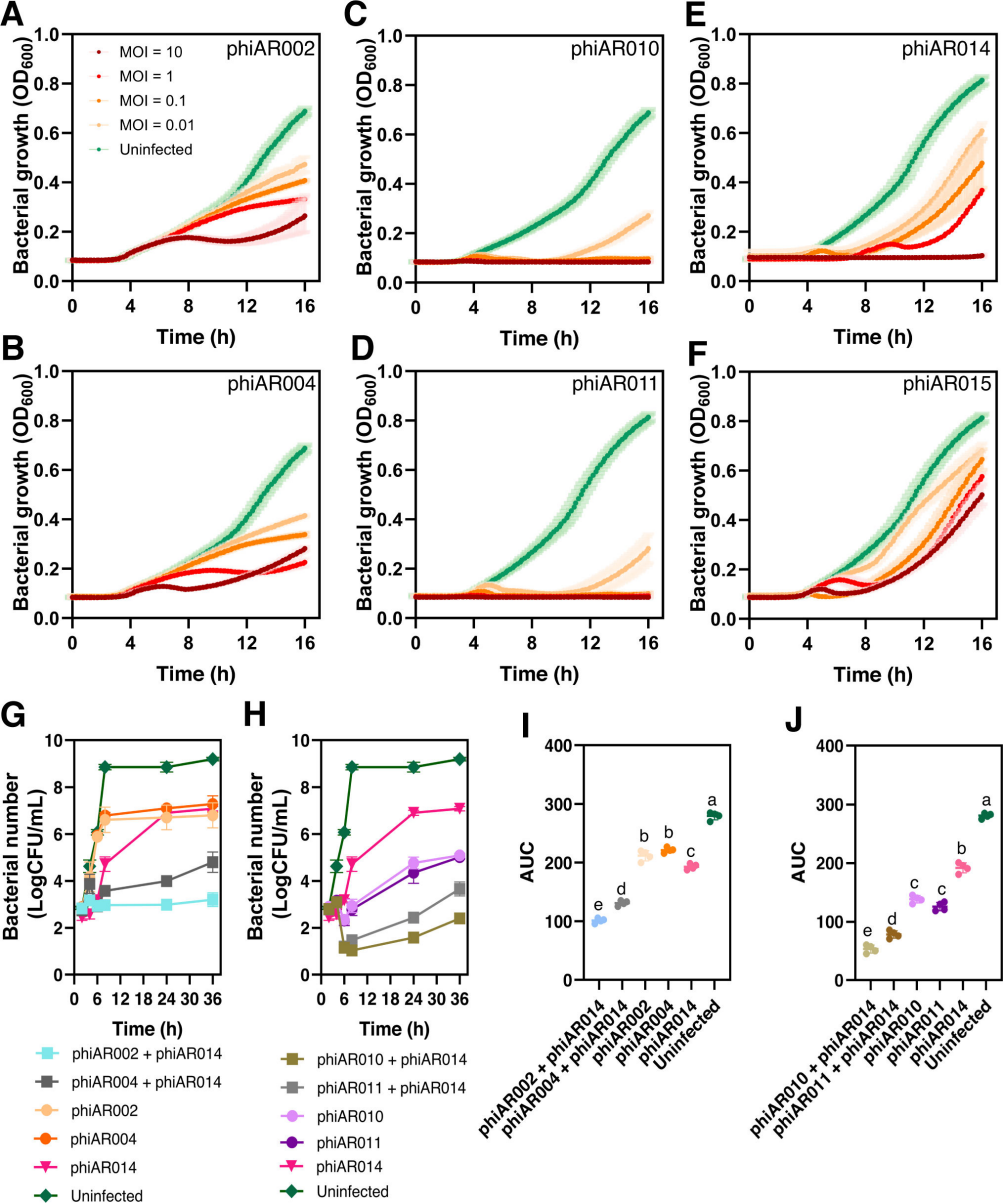
Impacts of varying multiplicity of infection (MOI) against *A. baumannii* parental host and phage killing ability of the phage cocktail composed of non-halo phage (SCP or MCP) and HHP. (**A–F**) Growth curve analysis of the parental bacteria in the presence of each phage (**A**) phiAR002 (SCP), (**B**) phiAR004 (SCP), (**C**) phiAR010 (MCP), (**D**) phiAR011 (MCP), (**E**) phiAR014 (HHP), and (**F**) phiAR015 (HHP). Log-phase bacteria were added to 96-well plates and infected with each phage at four MOIs: 0.01, 0.1, 1, and 10. The bacterial growth (OD_600_) was measured every 10 min for 16 h at 37°C. Averages of the growth curve are graphed, with the standard deviation. (**G–H**) Efficacy of phage cocktails composed of HHP with either SCP (**G**) or MCP (**H**) against the parental strain of *A. baumannii* was evaluated at a MOI of 1. In the cocktail treatments, two phages were combined at an equal ratio (0.5:0.5) and assessed at 0, 2, 4, 6, 8, and 24 h. The treatments included single-phage infections (MOI = 1) and combined phage preparations (0.5:0.5 ratio), consisting of four cocktails: (i) phiAR002 + phiAR014, (ii) phiAR004 + phiAR014, (iii) phiAR010 + phiAR014, and (iv) phiAR011 + phiAR014. Single-phage infections were performed at an MOI of 1. The bacterial growth revival was quantified at different time intervals for 36 h. (I–J) The area under the curve (AUC) of bacterial growth, calculated using GraphPad Prism, represents the main effects of phage cocktails composed of HHP in combination with either (**I**) SCP or (**J**) MCP. The data represent the mean ± standard deviation of at least three independent biological replicates. Statistical analysis in panels **I and J** was performed using one-way ANOVA followed by Tukey’s HSD test for multiple comparisons. Different letters above groups indicate significant differences (*P* ≤ 0.05).

We sought to determine whether variations in phage morphology influence the degree of bactericidal efficacy in suppressing bacterial growth. The MCP exhibited greater suppression of bacterial growth than the other phage groups (SCP and HHP) (Fig. 2A through F). In cases of SCP and HHP, the inhibitory effect improved with an increase in the number of phages. Bacterial growth was inhibited by the SCP phages (phiAR002 and phiAR004) at a multiplicity of infection (MOI) of 10 for approximately the first 9 h, followed by a subsequent resurgence in growth (Fig. 2A and B). The MCP phages (phiAR010 and phiAR011) at MOIs ranging from 0.1 to 10 demonstrated a prolonged ability to inhibit bacterial growth compared to an MOI of 0.01, effectively suppressing bacterial growth for up to 12 h before regrowth was observed (Fig. 2C and D). For HHP phages, phiAR014 at a high MOI of 10 suppressed bacterial growth over the entire observation period, whereas phiAR015 inhibited growth only during the initial 8 h, followed by bacterial regrowth (Fig. 2E and F). These findings suggest that, under the conditions tested, phages producing larger plaque sizes (MCP) exhibited greater bactericidal efficacy against the parental strain, as comparable growth patterns were observed across different MOI levels.

To expand the host range of the phages and enhance their killing ability, we combined two phages based on their host range spectra, which demonstrated lytic activity against distinct CRAB isolates from Phatthalung Hospital and Songklanagarind Hospital (Fig. 1C), as well as their plaque morphology. Furthermore, we hypothesized that combining halo plaques (HHP) with non-halo plaques (SCP and MCP), where the former suggests the presence of enzymes capable of degrading bacterial capsular polysaccharides (28, 29), could more effectively inhibit bacterial growth. This is based on the rationale that a phage cocktail comprising phages with diverse infection mechanisms may enhance overall bactericidal efficacy (24). For further investigation, we performed growth curve analysis using the parental host strain treated with phage cocktails comprising the HHP phage phiAR014 in combination with either an SCP phage or an MCP phage, evaluated under both combined and individual treatment settings. The results demonstrated that the phage cocktail comprising HHP combined with either an SCP or an MCP phage was more effective in suppressing bacterial growth over an extended period (36 h) than individual phage treatments at an MOI of 1 (Fig. 2G and H). The area under the curve (AUC) for the uninfected parental bacterial strain was significantly higher than that for all other treatment conditions (Fig. 2I and J). Meanwhile, the AUC for phage cocktails, specifically phiAR002 + phiAR014 and phiAR010 + phiAR014, was significantly reduced compared to all other conditions (*P* ≤ 0.05). The synergy of the phage cocktails was evaluated using the AUC and fractional inhibition (effect) at an MOI of 1. The combined phage resulted in a marked AUC reduction, with an observed effect ranging from 0.997 to 0.998, compared to the expected effect of 0.447 to 0.683, indicating a synergistic interaction. Among the combined phage treatments, the AUC varied significantly, with phiAR002 + phiAR014 and phiAR010 + phiAR014 demonstrating the most pronounced reduction (*P* ≤ 0.05). In contrast, treatment with either the SCP or MCP phage alone resulted in a reduction that was not statistically significant. These results highlight that phage formulations strategically combining phages with distinct plaque morphologies and bactericidal efficacy offer an effective approach to inhibit *A. baumannii* growth and delay bacterial regrowth over time.

### Biological characterization of phages phiAR002, phiAR010, and phiAR014, along with single-cell imaging, demonstrated phage-induced morphological changes in *A. baumannii* parental host

Among the isolated phages, phiAR002 (SCP), phiAR010 (MCP), and phiAR014 (HHP) were selected for investigation of their biological features based on three key criteria. First, they display distinct plaque morphologies and relatively wide but distinct host ranges (Fig. 1B and C). Secondly, each phage exhibited effective lytic activity against the parental bacterial strain and was capable of suppressing the development of phage-resistant populations (Fig. 2A through F). Third, when combined with the HHP phage phiAR014, the phage cocktail achieved a substantial bacterial reduction of ~5–6 log₁₀ CFU/mL, demonstrating superior efficacy compared to individual phages and other phage combinations (Fig. 2G through J).

**Fig 3:**
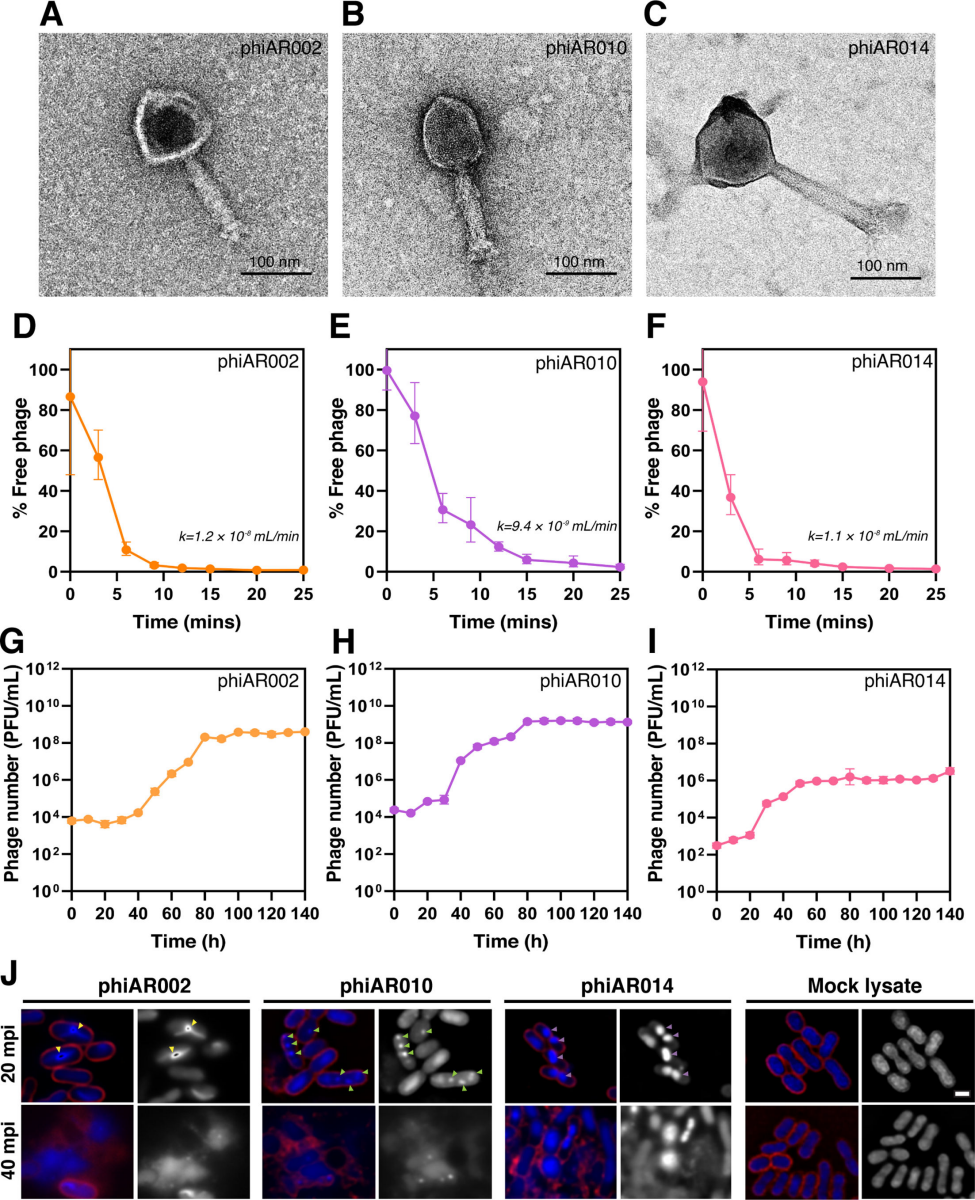
Phage morphology under transmission electron micrographs, along with a comparison of their growth dynamics and replication mechanisms during infection at the single-cell level. (**A–C**) TEM micrographs of negatively stained high-titer phage lysate (>10^8^ PFU/mL) samples, including phiAR002 (**A**), phiAR010 (**B**), and phiAR014 (**C**). Scale bars represent 100 nm. (**D–F**) Adsorption: Phage adsorption efficiency in TSB was measured over time (phiAR002 (**D**), phiAR010 (**E**), and phiAR014 (**F**)). The adsorption rate constant (*k*) was calculated using the equation $k=\frac{2.3}{Bt}log\left(\frac{P_{0}}{P}\right)$, where *k* is the adsorption rate constant (mL/min), *B* is the bacterial concentration, *t* is the time interval, *P₀* is the initial phage titer, and *P* is the unadsorbed phage titer at time *t*. (**G–I**) One-step growth curve: latent and burst phases of phages were analyzed (phiAR002 (**G**), phiAR010 (**H**), and phiAR014 (**I**)). Data are presented as the mean plus standard deviation of three independent biological replicates. (**J**) Time series imaging over a 40-minute period was performed on *A. baumannii* cultures treated with either phage phiAR002 (left panel; yellow arrows indicate toroidal-shaped DNA), phiAR010 (middle panel; green arrows indicate DAPI-stained puncta), phiAR014 (middle panel: purple arrows indicate the condensed DNA), or a mock lysate as a control (right panel). Bacterial cells at an optical density of 0.4 at 600 nm were infected with a multiplicity of infection (MOI) of 10 at 20- and 40-minute post-infection (mpi). Cells were stained with FM4–64 to visualize the cell membrane (red) and DAPI to label the nucleoid (blue or gray). The scale bar represents 1 µm.

Transmission electron microscopy analysis demonstrated that all selected phages were classified within the *Caudoviricetes* class and exhibited morphotypes characteristic of myoviruses, which are typically characterized by icosahedral heads and contractile tails (30). The phages displayed distinct head morphologies (Fig. 3A through C). Phage phiAR002 possessed capsids measuring ~77 ± 7 nm in width and 82 ± 10 nm in length, with contractile tails measuring 127 ± 6 nm in length and 30 ± 5 nm in width (Fig. 3A). Phage phiAR010 exhibited capsids measuring ~75 ± 2 nm in width and 102 ± 3 nm in length, with contractile tails measuring 128 ± 10 nm in length and 29 ± 5 nm in width (Fig. 3B). Phage phiAR014 displayed capsids measuring ~112 ± 3 nm in width and 98 ± 8 nm in length, with contractile tails measuring 177 ± 21 nm in length and 38 ± 8 nm in width (Fig. 3C).

Regarding phage adsorption ability, the majority of phiAR002 (~90%; Fig. 3D) and phiAR014 (~90%; Fig. 3F) adsorbed to the parental host within 6 min, with adsorption rate constants of 1.2 × 10⁻⁸ mL/min and 1.1 × 10⁻⁸ mL/min, respectively. In contrast, phiAR010 (~90%) required up to 12 min to achieve comparable adsorption (Fig. 3E) and exhibited a lower adsorption rate constant of 9.4 × 10⁻⁹ mL/min. Phages phiAR002 and phiAR010 exhibited a latent period of 30 min, followed by an extended lysis phase lasting 50 min, reaching a plateau after 80 min (Fig. 3G and H). The average burst size of phiAR002 and phiAR010 was approximately 678 and 882 phage particles per infected bacterial cell, respectively. While phage phiAR014 exhibited a latent period of 20 min, followed by a prolonged lysis phase of 30 min, reaching a plateau at 80 min, the average burst size was approximately 255 phage particles per infected bacterial cell.

To monitor the progression of the phage replication cycle, a single-cell infection assay was performed using DAPI staining to visualize phage DNA within host cells (24, 31). Morphological alterations in bacterial cells during phage infection can serve as indicators of the replication strategy, referred to as the mechanism of pre-killing (MOK) (24, 31, 32). Additionally, bacterial cytological profiling (BCP), a fluorescence microscopy-based approach, enables the identification of bacterial biosynthetic pathways targeted by antimicrobial agents through the analysis of characteristic morphological changes (33). The results revealed that *A. baumannii* cells undergoing phage infection exhibited notable morphological alterations over time in contrast to uninfected control cells, suggesting that these changes were induced by phage infection (Fig. 3J). Notably, infection with phiAR002 resulted in the appearance of toroidal-shaped DNA at 20 min post-infection (mpi) (Fig. 3J; yellow arrows), a hallmark typically associated with the action of protein translation inhibitors (31, 34). This observation aligns with known cytological profiles of *A. baumannii*, where protein synthesis inhibitors such as tetracycline, tigecycline, and minocycline bind directly to the ribosome, thereby disrupting translation and inducing the formation of toroidal DNA structures (34–37). However, during phiAR010 infection, multiple DAPI-stained puncta were uniformly distributed throughout the cells at 20 mpi (Fig. 3J; green arrows), whereas cells infected with phiAR014 displayed a single condensed nucleoid located near the mid-cell region at 20 mpi (Fig. 3J; purple arrows). These results indicate that each phage induces distinct morphological alterations in infected cells, suggesting the involvement of different MOKs during infection.

### Genomic characteristics of selected phages reveal significant genetic divergence among them

**Fig 4:**
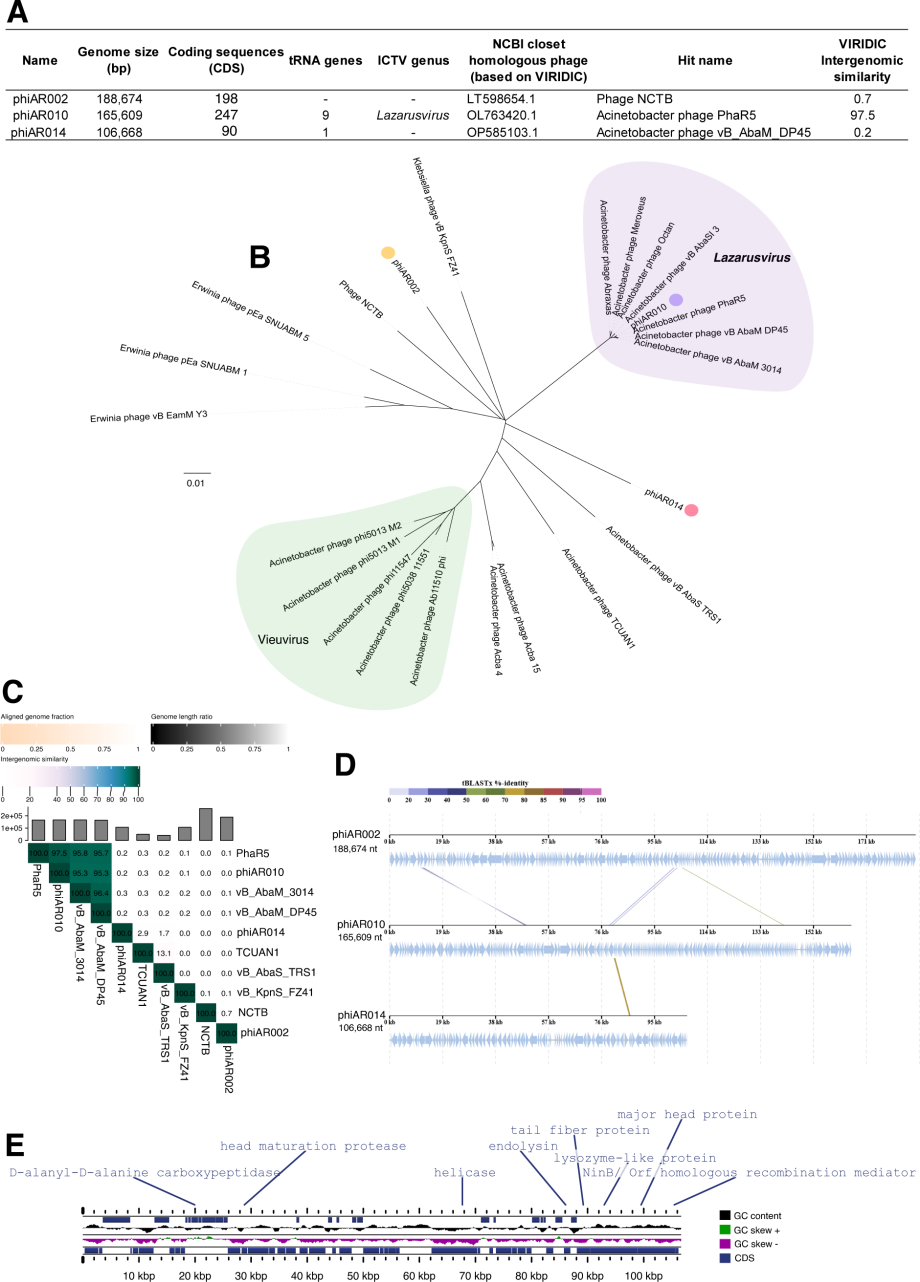
Phages phiAR002 (SCP), phiAR010 (MCP), and phiAR014 (HHP) exhibit genetic divergence. (**A**) The phage isolates selected in this study. (**B**) A phylogenetic tree based on whole-genome sequences was constructed for phages phiAR002 (orange dot), phiAR010 (purple dot), and phiAR014 (pink dot), along with other related phages. This tree was generated using the Genome-BLAST Distance Phylogeny (GBDP) approach via the VICTOR web service, incorporating 100 bootstrap replicates, and visualized using iTOL. The tree includes representatives from the *Lazarusvirus* (purple) and *Vieuvirus* (green) genera. (**C**) A VIRIDIC heatmap illustrates the intergenomic similarities among phages phiAR002, phiAR010, phiAR014, and related phages. The values indicate the percentage similarity between genome pairs. (**D**) Comparative genomic analysis of the three phages shows the arrangement and orientation of predicted coding sequences, with arrows representing genes and shaded lines indicating levels of sequence homology. (**E**) The genome of phage phiAR014 encodes enzymes potentially involved in bacterial cell wall degradation, including carboxypeptidase and lysozyme-like proteins.

Phages phiAR002, phiAR010, and phiAR014 contained double-stranded DNA genomes of 188,674 bp, 165,609 bp, and 106,668 bp in length, with GC contents of 39.8%, 36.7%, and 42.5%, respectively (Fig. 4A). MegaBLAST analysis revealed that only phage phiAR010 shares a high sequence similarity (99%) with Acinetobacter phage PhaR5, whereas phages phiAR002 and phiAR014 exhibited low sequence similarity to any known phages in current databases (Fig. 4A). Phylogenetic analysis using the Genome-BLAST Distance Phylogeny (GBDP) method via the VICTOR web service, together with intergenomic similarity assessments performed using Virus Intergenomic Distance Calculator (VIRIDIC), confirmed that phiAR010 was closely related to other members of the genus *Lazarusvirus* (Fig. 4B and C). Further comparative analysis of the genome organization and evolutionary relationships among the three phages demonstrated significant genetic divergence, supporting the distinct evolutionary lineages of phiAR002, phiAR010, and phiAR014 (Fig. 4C and D).

To further predict phage protein functions, annotated proteins from all phages, based on analyses using the PHASTER and GenBank databases, were found to be involved in DNA replication, nucleotide metabolism, host cell lysis, structural assembly, and auxiliary metabolic genes (AMGs). Firstly, phage phiAR002 encoded 198 open reading frames (ORFs) (Fig. 4A; Table S1), of which ~71% of ORFs encoded hypothetical proteins with unknown functions, and 58 ORFs were functionally annotated. The annotated proteins were involved in DNA replication and nucleotide metabolism, including single-stranded DNA binding protein (ORF 1), DNA topoisomerase (ORF 46 and ORF 177), DNA helicase (ORF 165 and ORF 178), and HNH endonuclease (ORF 99 and 163). The structural proteins of phiAR002 are key components of virion architecture, including major capsid proteins (ORF94), tail-associated proteins (ORF33, ORF34, ORF80, and ORF84), and DNA packaging proteins (ORF180 and ORF182). Furthermore, phiAR002 carries several auxiliary metabolic genes (AMGs) such as QueC-like queuosine biosynthesis proteins (ORF130 and ORF131), a QueE-like radical SAM domain protein (ORF169), and a DarB antirestriction protein (ORF133), which play roles in counteracting host defense mechanisms, particularly by protecting phage genomic DNA from degradation by bacterial restriction enzymes (38, 39).

Second, phage phiAR010 was classified within the genus *Lazarusvirus*, having satisfied the species-level threshold of 95%, as determined by intergenomic similarity analysis conducted using VIRIDIC (Fig. 4A and B). Phage phiAR010 encodes 247 open reading frames (ORFs) (Fig. 4A; Table S2), ~54% of which correspond to hypothetical proteins with unknown functions. The majority of annotated proteins were associated with DNA replication and nucleotide metabolism (~17%), tail assembly (~10%), and head formation and DNA packaging (~7%). Additionally, phiAR010 encodes proteins involved in host cell lysis, including holin (ORF76) and endolysin (ORF97), which facilitate the degradation of the peptidoglycan layer. Additionally, this phage encodes RNA polymerase-associated proteins (ORF68, ORF184, and ORF194) within its genome, indicating the potential for host-independent RNA transcription. Nine tRNA-encoding genes were identified using tRNAScan-SE (Table S3). However, the functional role of these tRNAs within the phage genome remains unclear, as the translation of viral proteins predominantly relies on host-derived tRNAs (40).

Thirdly, phage phiAR014 encodes 90 open reading frames (ORFs) (Fig. 4A; Table S4), the majority of which (approximately 85%) correspond to hypothetical proteins with unknown functions. Only 19 ORFs were functionally annotated and associated with DNA replication and nucleotide metabolism (ORF53, ORF77, and ORF90), host cell lysis (ORF17, ORF69, and ORF75), tail proteins (ORF73), head and packaging proteins (ORF25, ORF26, ORF81, ORF84, ORF86, ORF87, and ORF88), and tRNA-encoding genes using tRNAScan-SE. Furthermore, phiAR014 encodes peptidoglycan-degrading enzymes, commonly referred to as autolysins (Fig. 4E), including D-alanyl-D-alanine carboxypeptidase (ORF22), which cleaves terminal D-Ala residues at the fifth position of pentapeptides in peptidoglycans, resulting in cell death (41, 42). Additionally, phiAR014 encodes a lysozyme-like protein (ORF75) that functions as a glycoside hydrolase (IPR002196), involved in the hydrolysis of β-1,4-linked polysaccharide bonds between *N*-acetyl-D-glucosamine and *N*-acetylmuramic acid within the peptidoglycan heteropolymers of prokaryotic cell walls (15).

### Phage cocktails combining a HHP phiAR003 with either a SCP phiAR002 or MCP phiAR010 effectively suppressed co-infection by *A. baumannii* and *S. aureus* in both planktonic and biofilm states.

It is well known that the appearance of a halo surrounding plaques (HHP; phiAR014) formed by individual phages indicates the expression of functional depolymerases, which are typically associated with depolymerase activity and tail-associated phage proteins (15). Here, we hypothesized that these phages are capable of recognizing and degrading bacterial polysaccharides, thereby enhancing the suppression of co-infections caused by *A. baumannii* and *S. aureus*. We evaluated the effect of the HHP (phiAR014), in comparison with the non-halo phages phiAR002 (SCP) and phiAR010 (MCP), on antibacterial activity against polymicrobial infections caused by *A. baumannii* and *S. aureus*, including both planktonic cells and biofilm formation.

**Fig 5:**
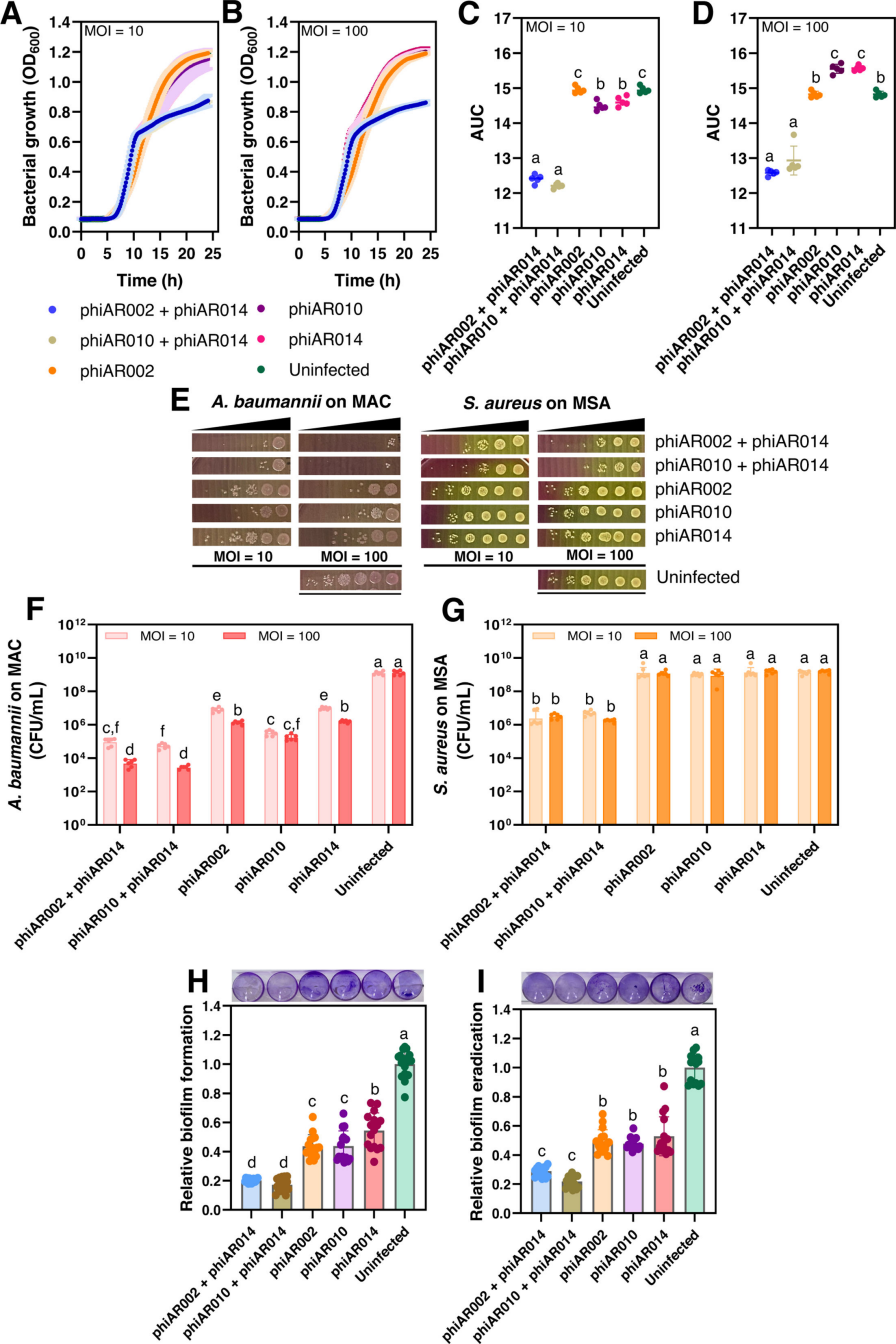
The combination of a HHP with either a SCP or a MCP phage demonstrates potential in suppressing co-infection by *A. baumannii* and *S. aureus* in both planktonic cultures and during biofilm formation and eradication. (**A and B**) Bacterial growth inhibition, as measured by optical density at 600 nm, was assessed using a phage cocktail comprising a halo-forming phage in combination with either a SCP or a MCP at MOIs of 10 (**A**) and 100 (**B**). Log-phase cultures (1 × 10⁶ CFU/mL) of *A. baumannii* and *S. aureus* (0.5 × 10^5^ CFU/mL each) were prepared in TSB. The cultures were infected with each phage cocktail at MOIs of 10 (0.5 × 10⁶ PFU/mL: 0.5 × 10⁶ PFU/mL) and 100 (0.5 × 10⁷ PFU/mL: 0.5 × 10⁷ PFU/mL). Single-phage infections were performed at MOIs of 10 (1 × 10⁷ PFU/mL) and 100 (1 × 10⁸ PFU/mL). (**C and D**) Area under the curve (AUC) measurements over different phage cocktails and individual phage at MOIs of 10 (**C**) and 100 (**D**). The data in panels **A–D** represent the mean ± standard deviation of six independent biological replicates. (**E–G**) The number of surviving bacterial colonies was determined via dilution plate counting on MacConkey agar (Mac) for *A. baumannii* and mannitol salt agar (MSA) for *S. aureus* (**E**). The results indicate that the phage cocktail effectively suppresses co-infection by *A. baumannii* and *S. aureus* when infected at MOIs of 10 (**F**) and 100 (**G**), compared to individual normal plaque-forming phages. Each data point represents an independent biological replicate (*n* = 6), with means presented as box plots. Biofilm prevention (**H**) and removal (**I**) activities were evaluated using both phage cocktails and individual phage treatments at a final concentration of 1 × 10⁸ PFU/mL. Biofilm biomass was quantified by crystal violet staining (OD_570_). Bacterial suspensions (1 × 10⁶ CFU/mL), consisting of *A. baumannii* (0.5 × 10⁵ CFU/mL) and *S. aureus* (0.5 × 10⁵ CFU/mL), were prepared in TSB supplemented with 2.5% glucose. Phage cocktails were subsequently added at a final concentration of 1 × 10⁸ PFU/mL, including (i) phiAR002 (0.5 × 10⁷ PFU/mL) + phiAR014 (0.5 × 10⁷ PFU/mL) and (ii) phiAR010 (0.5 × 10⁷ PFU/mL) + phiAR014 (0.5 × 10⁷ PFU/mL). The combined halo-forming phage cocktails exhibited relatively lower efficacy compared with individual phage treatments. Each data point represents an independent biological replicate (*n* = 16), with averages depicted as boxplots. Statistical analysis in panels **C– D and F– I** was performed using one-way ANOVA followed by Tukey’s HSD post hoc test for multiple comparisons. Different letters above groups indicate significant differences (*P* ≤ 0.05).

To investigate this further, we first performed a killing assay of the phage cocktail in the co-culture and compared it with individual phages. The results indicated that the HHP phiAR014 combined with either a SCP phiAR002 or MCP phiAR010 could produce a synergistic bacterial suppression effect over 24 h compared to individual phages at MOIs of 10 and 100 (Fig. 5A through E), as measured by the AUC. A synergistic effect was observed within approximately 10 h and persisted throughout the 24-hour observation period at MOIs of 10 (Fig. 5A) and 100 (Fig. 5B). Co-cultivation of *A. baumannii* and *S. aureus* was significantly inhibited by phage cocktails containing phiAR002 + phiAR014 and phiAR002 + phiAR014 (Fig. 5C and D) compared to individual phages and uninfected cells (*P* ≤ 0.05). However, these cocktails did not demonstrate significant inhibitory effects regardless of the MOI used. To further validate this inhibitory effect, the presence of *A. baumannii* and *S. aureus* was assessed by enumeration at 24 h on MAC and MSA agar, respectively (Fig. 5E). The overall trend of inhibitory activity was similar in the killing assay, although the phage cocktail exhibited lower suppression efficiency against *A. baumannii* than *against S. aureus*. In the co-cultivation setting, treatment with the phage cocktail at an MOI of 100 resulted in a significant reduction of *A. baumannii* by ~2 log₁₀ CFU/mL compared to individual phage treatment, and by approximately 5 log_₁₀_ CFU/mL relative to the uninfected control (Fig. 5F). Of note, both phage cocktails exhibited a synergistic antibacterial effect against *S. aureus*, leading to a significant reduction of approximately 2 log_₁₀_ CFU/mL compared to the individual phage treatments and the uninfected control (Fig. 5G).

To evaluate the effects of phage cocktails on biofilm formation during the co-cultivation of *A. baumannii* and *S. aureus*, biofilm inhibition and eradication assays were conducted (Fig. 5H and I). In these experiments, phage titers (1 × 10⁸ PFU/mL) were applied based on the initial bacterial inoculum (1 × 10^6^ CFU/mL). Accordingly, while the reported phage titer accurately reflects the conditions used in the biofilm inhibition assay (Fig. 5H), the effective phage-to-bacteria ratio during the biofilm eradication assay (Fig. 5I) is expected to be lower due to the substantially increased bacterial population within mature biofilms. Despite this, the results demonstrated a significant reduction in biofilm formation (Fig. 5H) and enhanced biofilm clearance (Fig. 5I) following treatment with phage cocktails compared with individual phage treatments. The individual phages phiAR002, phiAR010, and phiAR014 showed comparable efficacy in biofilm eradication (Fig. 5I). In contrast, phiAR002 and phiAR010 exhibited significantly greater inhibition of biofilm formation than phiAR014 (Fig. 5H). The potential synergistic effects of the phage cocktails were further evaluated using relative biofilm formation and fractional inhibition. Phage cocktails resulted in a marked reduction in both relative biofilm formation and biofilm biomass during eradication assays, with observed effects ranging from 0.712 to 0.828 compared with the expected effect of 0.709–0.85, suggesting a synergistic interaction. These findings highlight the enhanced efficacy of phage cocktails comprising a halo-plaque–forming phage in combination with other lytic phages and support their potential as a therapeutic strategy for targeting polymicrobial infections caused by *A. baumannii* and *S. aureus* in both planktonic and biofilm-associated states.

## DISCUSSION

Carbapenem-resistant *A. baumannii* (CRAB) has consistently emerged as a significant cause of nosocomial infections, presenting a significant challenge to public health because of its resistance to last-line antibiotics (43, 44). In response to this growing concern, phages have been recognized as among the most promising agents to replace or be used in combination with conventional antibiotics. As *A. baumannii* employs a range of defense mechanisms against phage infection, resulting in the development of phage resistance (45, 46), the use of biologically and genetically diverse phages in a cocktail can enhance antibacterial efficacy (24). This is because different phages may recognize distinct bacterial receptors, making it difficult for the bacterial host to simultaneously develop resistance to the cocktail. Another important consideration is that CRAB outbreaks often involve lineages that differ from those circulating in other geographic regions (23, 27). Therefore, employing a combination of phages with broader host ranges is especially valuable, as they can target and eliminate a wider array of pathogenic strains and potentially related species, which is critical for developing effective, region-specific infection prevention strategies. In this study, we selected phages targeting CRAB based on their plaque morphology, classified as small clear (SCP), medium clear (MCP), or hazy-halo (HHP) plaques (Fig. 1A and B), and host range spectrum. These phages, representing distinct plaque types, demonstrated the ability to lyse a broad spectrum of CRAB strains isolated from secondary and tertiary care hospitals across various geographical regions in Southern Thailand (27) (Fig. 1C; Fig. S1A and B). To broaden the host range, phages were combined based on differences in plaque morphology and antimicrobial activity. As anticipated, phage combinations comprising diverse phage types were more effective in suppressing bacterial growth for up to 36 h than individual phage treatments (Fig. 2A through J). The most effective combination, consisting of phages phiAR002, phiAR010, and phiAR014, likely exhibited enhanced antibacterial activity because of their distinct mechanisms of action and genetic backgrounds, as suggested by their infectivity profiles against various CRAB lineages based on molecular epidemiological analyses (Fig. 1C; Fig. S1B). Host range is a fundamental characteristic in the study of phage biology and is determined by a series of molecular interactions between the phage and its host throughout the infection cycle. These interactions provide insights into the molecular mechanisms that facilitate phage infection in multiple hosts (47). Importantly, the host range of phages is not a static characteristic; it can evolve over time and exhibit considerable plasticity (48). Moreover, given the wide geographical variation in the distribution of CRAB determinants (23), our phages show significant potential for therapeutic applications in the management of region-specific infections.

As mentioned previously, a cocktail composed of phages exhibiting diverse biological characteristics, including distinct differences in growth dynamics, replication mechanisms, genome sizes, and host range spectra, can enhance antimicrobial efficacy. Within our phage cocktail, the observed changes in bacterial morphology during phage infection could be attributed to phage replication or host takeover mechanisms (31, 49). These morphological changes provide insight into the intracellular replication dynamics of phages. For example, the BCP technique was employed to investigate the metabolic pathway targeted by the phage Seahorse during infection, indicating that the phage likely inhibits protein translation in *Vibrio parahaemolyticus* at an early stage in its lytic cycle (31). This finding is consistent with the strategy employed by one of our phages, phiAR002 (Fig. 2J), which may involve hijacking the host machinery, specifically replication, transcription, and translation, to suppress host defenses and prioritize phage protein synthesis. Phage phiAR010 may exhibit a lower degree of replication factory organization, as evidenced by the presence of numerous uniformly distributed DAPI-stained puncta, similar to *Pseudomonas* phage Callisto (49), *Bacillus* phage SPP1 (50), and *Escherichia coli* phage lambda (51), and appears to organize its replisomes in a manner that allows replication to proceed without confinement within defined cellular boundaries. In contrast to phiAR014, which displays a single condensed DAPI-stained DNA, the observed phenotypes strongly suggest that all three phages utilize distinct replication mechanisms during infection. Further investigations are required to elucidate whether divergent host machinery hijacking by these phages occurs during the infection cycle. Although the precise causes of variation in replication mechanisms among the three phages remain unclear, genetic divergence is evident. Consequently, the selection of phages with distant phylogenetic relationships may contribute to an enhanced overall bactericidal efficacy. The phages in our cocktail displayed significant variations in genome size. Notably, phiAR002 (SCP), phiAR010 (MCP), and phiAR014 (HHP) each possessed relatively large genomes, ranging from approximately 106 to 188 kbp (Fig. 4A). Conversely, most *Acinetobacter* phages possess relatively small genomes, typically ranging from 40 to 90 kbp, with the exception of phage vB_AbaM_ME3, which exhibits a notably large genome size of approximately 234 kbp (16). Accordingly, only phiAR010 could be classified within the *Lazarusvirus* genus (Fig. 4A and B), which is the main genus of the *Straboviridae* family (16), whereas phiAR002 and phiAR014 showed limited associations with other families in the NCBI database. Phylogenetic analysis indicated that these phages clustered into distinct clades (Fig. 4B and D), reflecting their diverse evolutionary origins, which may have contributed to overcoming phage-resistant bacterial strains (Fig. 3G and H). In the clinical setting, phage cocktails targeting multiple pathogens may enhance treatment efficacy by selecting phages that minimize cross-reactivity and interference (46). Genomic analysis underscores the importance of whole-genome sequencing alongside traditional host range and inferred replication mechanisms identified using the BCP technique. Incorporating genetically and phenotypically distinct phages into cocktails can minimize redundancy and enhance the efficiency of phage production and purification, thereby increasing the feasibility of phage therapy.

Diverse *A. baumannii* phages exhibit distinct structural and evolutionary features within functional domains associated with depolymerase activity and tail-associated proteins (16). These phages typically encode tail fiber and spike proteins critical for host recognition, with some possessing depolymerase functions that facilitate bacterial cell wall degradation and enhance the efficiency of infection (15). In this study, the identification of a tail fiber protein with putative glycoside hydrolase or lysozyme activity (Fig. 4E), along with the presence of halo zones in the plaques formed by HHP phiAR014, gives rise to the possibility that the phage might possess the capability to depolymerize exopolysaccharides and disrupt biofilms (52, 53). This characteristic indicated a potential anti-biofilm effect, which may be beneficial for addressing polymicrobial infections involving *A. baumannii* and *S. aureus*. Such co-infections are of particular concern in healthcare settings, particularly among intensive care unit patients and individuals with compromised immune systems (1, 54). Biofilm formation is a key virulence factor that enables these pathogens to adhere, colonize, and persist in clinical settings (54). In addition to the direct application of HHP phiAR014 within phage cocktails, it has been proven to be effective in targeting planktonic cells and disrupting bacterial biofilms in polymicrobial infections (Fig. 5A through I). Our results indicated that cocktails comprising HHP phiAR014 combined with either SCP phiAR002 or MCP phiAR010 exhibited a synergistic bactericidal effect over 24 h at MOIs of 10 and 100, with inhibition observed within 10 h (Fig. 5A through D). Surprisingly, these cocktails significantly reduced bacterial loads compared to individual phages and controls, achieving approximately a 2 log₁₀ CFU/mL reduction in *A. baumannii* and *S. aureus* at MOI 100, and up to a 5 log₁₀ CFU/mL reduction in *A. baumannii* relative to untreated controls (Fig. 5E through G). Furthermore, biofilm inhibition and eradication assays demonstrated that phage cocktails markedly decreased biofilm formation and enhanced biofilm clearance compared to single-phage treatments (Fig. 5H through I). Synergistic interactions within the phage cocktails were confirmed through analyses of relative biofilm formation, indicating efficacy beyond the additive effects. These findings highlight the superior antibacterial and anti-biofilm potentials of phage cocktails that include halo-plaque morphology, supporting their potential as therapeutic agents against polymicrobial infections involving *A. baumannii* and *S. aureus* in both planktonic and biofilm-associated states. The evidence suggests that the phage cocktail containing the halo-forming phage phiAR014 harbors two enzymes that contribute to bacterial cell wall hydrolysis. These include a lysozyme-like protein (ORF75), capable of cleaving N-acetylglucosamine (NAG) and N-acetylmuramic acid (NAM), which are the principal structural components of the peptidoglycan layer in both Gram-positive and Gram-negative bacteria (55), and a carboxypeptidase enzyme (ORF22), typically associated with phages infecting Gram-positive bacteria (56), which facilitates cleavage within or between peptide chains linked to NAM. Collectively, these peptidoglycan-degrading enzymes might promote bacterial autolysis, thereby contributing to the inhibition of polymicrobial populations. Nevertheless, further investigation is required to obtain direct evidence confirming that the observed synergistic inhibition of *A. baumannii* and *S. aureus* is attributable to the activity of depolymerases or cell wall hydrolases encoded by the halo-forming phages.

In conclusion, a thorough understanding of phage biology and genetic diversity is essential for advancing novel phage therapies, particularly for addressing the increasing challenges posed by multidrug-resistant polymicrobial infections. This strategy plays a crucial role in improving the practicality of phage therapy by preventing redundancy and significantly reducing the time and resources required for phage production and purification. However, research on the cell wall hydrolases and mechanisms utilized by phages as potential alternative antibacterial agents is still in the early stages of development. Further investigations are required to explore their formulation and bioengineering potential for application in clinical settings.

## MATERIALS AND METHODS

### Bacterial strains and growth conditions

A total of 52 bacterial strains were used in this study, including 46 carbapenem-resistant *A. baumannii* (CRAB) strains isolated from Phatthalung Hospital (22 strains) and Songklanagarind Hospital (24 strains) (27), *A. baumannii* DMST 2071, *A. baumannii* ATCC 19606, *Klebsiella pneumoniae* ATCC 700603, *Staphylococcus aureus* ATCC 29312, *Pseudomonas aeruginosa* ATCC 27853, and *Escherichia coli* ATCC 25922. All bacterial strains were stored in trypticase soy broth (TSB) supplemented with 25% glycerol at −80°C. A single colony grown on trypticase soy agar (TSA) was inoculated into 5 mL of TSB and incubated overnight at 37°C with shaking at 150 rpm. Subsequently, the overnight culture was diluted 1:50 in fresh TSB and incubated under the same conditions until log growth phase was reached.

### Phylogenetic tree of clinical strains based on multilocus sequence typing (MLST) using the Oxford scheme

To further investigate the clonal relationships among *A. baumannii* isolates, phylogenetic trees were constructed based on the Oxford MLST scheme, either by comparison with reference databases or among the clinical CRAB isolates. The first phylogenetic tree included previously published genomes from our study (*n* = 48) alongside the *A. baumannii* ATCC 19606 reference genome. The second tree was constructed using the same set of genomes (*n* = 48), the ATCC 19606 genome (*n* = 1), and an additional 151 publicly available genomes obtained from the European Nucleotide Archive (ENA) database. These publicly sourced genomes originated from various countries, including Australia (*n* = 37), Bangladesh (*n* = 8), Brazil (*n* = 42), Ethiopia (*n* = 6), Greece (*n* = 1), India (*n* = 3), Italy (*n* = 1), the Netherlands (*n* = 12), Nigeria (*n* = 3), Pakistan (*n* = 2), Rwanda (*n* = 1), Singapore (*n* = 7), South Africa (*n* = 3), Sweden (*n* = 9), Switzerland (*n* = 6), Thailand (*n* = 1), the United Kingdom (*n* = 1), and Vietnam (*n* = 8). Briefly, the allele sequences were obtained from the PubMLST database and aligned with the CRAB genome using the BLASTN algorithm. The corresponding allele sequences were extracted and concatenated in the order defined by the Oxford MLST scheme. Multiple sequence alignments of all isolates were conducted, and phylogenetic trees were constructed using FastTree with the maximum likelihood method and 1,000 bootstrap replicates. The resulting trees were visualized using the Interactive Tree of Life online tool.

### Phage isolation, purification, plaque morphology, and propagation

Water and pig fecal samples were collected from Songkhla, Thailand. Briefly, water samples were filtered through a 0.45 µm membrane and mixed with TSB powder, while pig feces were combined with TSB in a 1:1 ratio. A log-phase culture of CRAB stain PT061 was then added to each mixture, followed by incubation at 37°C with shaking at 150 rpm for 24 h. After centrifugation, the supernatant was filtered and mixed with TSB containing 0.35% agar (TSA soft agar) before being combined with the host bacteria. This mixture was overlaid on TSA plates and incubated at 37°C for 24 h to observe plaque formation. Individual plaques were collected and subjected to at least three rounds of replating to ensure phage purification. The six purified phage stocks were suspended in SM buffer (200 mM NaCl, 10 mM MgSO4·7H2O, and 50 mM Tris-HCl, pH 7.5) and stored at 4°C. Plaque morphology, including size and other characteristics, was determined for the isolated phages.

For phage propagation, 200 µL of the host bacterial culture was combined with 50 µL of phage suspension and 5 mL of TSA soft agar. This mixture was then overlaid onto a TSA plate and incubated at 37°C for 24 h. Following incubation, 5 mL of SM buffer was added to the plate, which was then incubated at room temperature for 5–6 h. The bacterial cells were subsequently removed by centrifugation, and the resulting supernatant was filtered through a 0.45 µm membrane filter before being stored at 4°C for subsequent experiments. Phage titration was performed by mixing tenfold serial dilutions of phage stocks with the host bacterial culture and plating on TSA soft agar. The plates were incubated overnight at 37°C, after which phage quantities were determined by counting plaques and expressed as plaque-forming units (PFU) per milliliter.

### Phage lytic ability and time-kill kinetics of the combined phage cocktail of HHP either SCP or MCP

To assess the lytic activity of individual phages, killing curves were generated using a 96-well microplate by monitoring the growth of CRAB strain PT061. A log-phase bacterial suspension was added to each well, followed by the addition of phage lysates diluted in SM buffer to achieve MOIs of 0.01, 0.1, 1, and 10. Control wells containing the medium alone and bacterial cultures without phages were also included. The optical density at 600 nm was measured automatically at 10-minute intervals over an 18-h period and incubated at 37°C. All experiments were conducted independently in triplicates.

To develop the phage cocktail, phiAR014 was selected based on its lytic activity and subsequently combined with either SCP (phiAR002 and phiAR004) or MCP (phiAR010 and phiAR011). The CRAB strain PT061 was cultured to the log phase in TSB and infected at a multiplicity of infection (MOI) of 1 following incubation at 37°C. Treatments included single-phage infection (MOI = 1) or combined phage preparations at a 0.5:0.5 ratio, comprising four cocktails: (i) phiAR002 + phiAR014, (ii) phiAR004 + phiAR014, (iii) phiAR010 + phiAR014, and (iv) phiAR011 + phiAR014. Time-kill assays were performed by enumerating bacterial counts at 0, 2, 4, 6, 8, and 24 h post-infection using the agar dilution plate count method. The AUC was calculated, and statistical analyses were conducted using one-way analysis of variance (ANOVA) in GraphPad Prism. Post hoc testing was used to identify statistically significant differences between the conditions and phage treatments.

Phage cocktail synergy was assessed using a previously described method (57). Bacterial growth was quantified using area under the curve (AUC) data, which were used to calculate fractional inhibition as follows:


(1) $$E_{A}=1-\frac{B_{A}}{B_{0}},$$

where $B_{0}$ represents the no-phage control and $B_{A}$ represents treatment with phage A alone; similarly,


(2) $$E_{B}=1-\frac{B_{B}}{B_{0}},$$

where $B_{B}$ represents treatment with phage B alone.

The expected combined effect, assuming independent phage action, was calculated as:


(3) $$E_{expected}=E_{A}+E_{B}-\left(E_{A}\times E_{B}\right).$$

The observed combined effect was determined as:


(4) $$E_{observed}=1-\frac{B_{AB}}{B_{0}},$$

where $B_{AB}$ represents bacterial growth after combined treatment with phages A and B.

Synergy was interpreted as follows:

- $E_{observed}>E_{expected}$: synergistic interactions
- $E_{observed}\approx E_{expected}$: additive effect
- $E_{observed}<E_{expected}$: antagonistic interactions

### Phage biological characterization

#### Host range

A total of 52 bacterial strains were used to evaluate the host range, including 46 CRAB isolates: 22 from Phatthalung Hospital and 24 from Songklanagarind Hospital (27), *A. baumannii* DMST 2071, *A. baumannii* ATCC 19606, *Klebsiella pneumoniae* ATCC 700603, *Staphylococcus aureus* ATCC 29312, *Pseudomonas aeruginosa* ATCC 27853, and *Escherichia coli* ATCC 25922. A modified host range assay was performed using a 96-well microplate. Briefly, 10 µL of bacterial culture was mixed with 50 µL of TSB and 70 µL of 1% low-melt TSA, then allowed to solidify for 15 min. Subsequently, 50 µL of either phage solution or SM buffer (control) was added to each well, followed by incubation at 37°C for 24 h to observe the clear solution. All experiments were performed independently in triplicate.

#### Phage morphology by electron microscopy

Phage suspension was dropped onto the lawn of the parental host and incubated at 37°C for 24 h. The resulting clear zones were collected, immersed in SM buffer, and stored at 4°C overnight. Following centrifugation at 5,000 rpm for 5 min, the supernatant was filtered through a 0.45 µm membrane. The concentrated phage suspension was then applied to a copper grid and stained with 2% uranyl acetate solution for 2 min. After rinsing and blotting, grids were dried for 10 min. Phage morphology was examined using a Talos F200i transmission electron microscope (Thermo Fisher Scientific, Waltham, MA, USA) operating at 200 kV.

#### Phage adsorption

A phage adsorption curve was established to quantify the time required for phage attachment to host cells. Host cells were cultured to the logarithmic growth phase, followed by infection with phages at a MOI of 0.01 and incubation at 37°C. Aliquots of 300 µL were collected at 0, 3, 6, 9, 12, 15, 20, and 25 min and immediately filtered through a membrane with a pore size of 0.45 µm. The concentration of the unadsorbed phages in the filtrate was subsequently determined. All experiments were conducted independently in triplicates. Adsorption rate constant (*k*) was calculated following Kropinski’s protocol (58).

#### One-step growth curve

A one-step phage growth curve was constructed to assess the latent period and burst size of the phages. Five milliliters of the host culture in the logarithmic phase was centrifuged and resuspended in 1 mL of TSB. The parental host cells were infected with phage at a MOI of 0.01 and incubated at 37°C for 10 min to allow phage adsorption. Following centrifugation at 10,000 rpm for 1 min, the supernatant containing the free phages was collected to quantify the number of unadsorbed phages. This value was then subtracted from the initial phage count to estimate the number of infected cells. The pellet, containing the infected cells, was resuspended in 50 mL of TSB and incubated at 37°C. Samples were taken at 10-minute intervals over a period of 140 min and filtered through a 0.45 µm membrane. The phage titer was determined by a spot titer assay using the double-layer agar method. The latent period was defined as the duration from the initiation of the experiment to the onset of phage progeny production. The burst size was calculated by subtracting the number of unadsorbed phages from the average maximum phage yield, with the resulting value divided by the number of infected cells (PFU/CFU) (59). All experiments were performed independently in triplicate.

#### Single-cell infection assay

A single-cell assay was conducted to examine the phage-induced morphological changes (24, 49). The parental host culture was grown to an optical density at 600 nm of approximately 0.4 and subsequently infected with phages at a MOI of 10, followed by incubation at 37°C for 20 and 40 min. Infected cells were collected by centrifugation and stained with fluorescent dyes (2 μg/mL FM 4-64 and 2 μg/mL DAPI). After a subsequent centrifugation step, 3 μL of the cell suspension was applied onto an agarose pad (1.2% agarose in 20% LB medium). The samples were then observed under a ZEISS AxioObserver 7 microscope.

### Phage genome sequencing and analysis

High-titer phage lysates were concentrated using polyethylene glycol (PEG) precipitation. The lysate was combined with sodium chloride (NaCl) and PEG 8000 to final concentrations of 1 M and 10% (wt/vol), respectively. Following incubation overnight at 4°C, the mixture was centrifuged at 7,000 rpm for 30 min at 4°C. The supernatant was discarded, and the resulting pellet was air-dried prior to resuspension in deionized water. To remove contaminating bacterial genetic material, the purified phage preparation was treated with 2 units of DNase I and then incubated overnight at 37°C. Subsequently, 0.1 M EDTA was added to achieve a final concentration of 5 mM, and the mixture was incubated at 75°C for 10 min to inactivate the DNase. Phage capsids were then digested by incubating the sample with 10 mg/mL proteinase K and 0.5% sodium dodecyl sulfate (SDS) at 65°C for 1 h. Phage DNA was extracted using the phenol-chloroform-isoamyl alcohol method. Specifically, an equal volume of phenol-chloroform-isoamyl alcohol (25:24:1) was added to the lysed phage solution, and the mixture was vigorously vortexed. The solution was centrifuged at 10,000 rpm for 10 min, and the upper aqueous layer was transferred to a new microcentrifuge tube. The extraction step was repeated as required. DNA precipitation was achieved by adding 0.3 volumes of 3 M sodium acetate (CH₃COONa) and 1 volume of isopropanol, followed by gentle mixing and incubation at –20°C overnight. The precipitated DNA was pelleted by centrifugation at 10,000 rpm for 10 min. The supernatant was discarded, and the pellet was washed with 70% ethanol before centrifugation under the same conditions. After removing ethanol, the DNA pellet was air-dried until clear and subsequently dissolved in deionized water. The DNA quality was assessed by measuring the absorbance ratio at 260/280 nm, and the concentration was determined using a Nanodrop spectrophotometer. The purified DNA was stored at –20°C until further use.

Whole-genome sequencing was performed using the Illumina MiSeq platform. The quality of the sequencing reads was assessed using FASTQ, and assembly into contigs was performed using SPAdes version 3.11.1. Open reading frames (ORFs) were identified and analyzed using the Galaxy server and PHASTER, followed by annotation using BLASTp on the NCBI server. Intergenomic similarities among the related phages were evaluated using a VIRIDIC. A comparative analysis of multiple genomic loci was conducted using the DiGAlign online tool. Phylogenetic trees were constructed based on the Genome-BLAST Distance Phylogeny (GBDP) method, generated using the VICTOR web service with 100 bootstrap replicates, and subsequently visualized using Interactive Tree of Life online tool.

### Synergistic antibacterial effect of a combined phage cocktail against co-infection *A. baumannii* and *S. aureus* of planktonic and biofilm states

#### Phage killing assay

The bactericidal efficacy of the phage cocktails (i) phiAR002 + phiAR014 and (ii) phiAR010 + phiAR014 was evaluated against co-cultures of *A. baumannii* and *S. aureus* and compared with individual phage treatments and an untreated control. Briefly, log-phase bacterial cultures at 1 × 10⁶ CFU/mL (comprising *A. baumannii* at 0.5 × 10^5^ CFU/mL and *S. aureus* at 0.5 × 10^5^ CFU/mL) were prepared in TSB. Cultures were infected with each phage cocktail at MOIs of 10 (0.5 × 10⁶ PFU/mL : 0.5 × 10⁶ PFU/mL) and 100 (0.5 × 10⁷ PFU/mL : 0.5 × 10⁷ PFU/mL), whereas single-phage infections were conducted at MOIs of 10 (1 × 10⁷ PFU/mL) and 100 (1 × 10⁸ PFU/mL). Bacterial suspensions were added to 96-well microplates and mixed with the respective phage cocktails according to the designated MOIs following incubation at 37°C for 24 h. Bacterial growth was monitored by measuring optical density at 600 nm (OD₆₀₀) at 10-minute intervals over a 24-hour period. Viable bacterial counts were determined at 24 h using mannitol salt agar (MSA) and MacConkey agar dilution plates. All assays were performed independently in triplicates. The AUC was calculated, and statistical analysis was conducted using one-way ANOVA in GraphPad Prism, followed by post hoc tests to identify significant differences among the treatments. Phage cocktail synergy was assessed using the above-described method, in which bacterial growth was quantified based on area under the curve (AUC) data.

#### Biofilm inhibition and eradication assay

The effect of phage cocktails on biofilm formation by *A. baumannii* and *S. aureus* was assessed using a crystal violet assay. Briefly, bacterial suspensions at 1 × 10⁶ CFU/mL (comprising *A. baumannii* at 0.5 × 10^5^ CFU/mL and *S. aureus* at 0.5 × 10^5^ CFU/mL) were prepared in TSB supplemented with 2.5% glucose. Subsequently, phage cocktails at a final concentration of 1 × 10⁸ PFU/mL, consisting of (i) phiAR002 (0.5 × 10⁷ PFU/mL) + phiAR014 (0.5 × 10⁷ PFU/mL) and (ii) phiAR010 (0.5 × 10⁷ PFU/mL) + phiAR014 (0.5 × 10⁷ PFU/mL), were added. The cultures were incubated at 37°C for 48 h, alongside treatments with individual phages (1 × 10⁸ PFU/mL) and an untreated control. After incubation, tubes were gently washed twice with 0.85% normal saline solution to remove non-adherent cells and fixed with methanol for 15 min. Wells were stained with 0.1% crystal violet for 20 min, washed with 0.85% normal saline solution, and the dye was then solubilized with 200 µL of 95% ethanol. The absorbance was measured at 570 nm using a microplate spectrophotometer, and the remaining biofilm ratio was calculated.

Mature biofilms were established by incubating the bacterial suspensions described above at 37°C for 48 h. Subsequently, the biofilms were treated with either the phage cocktail or individual phages, as previously described, and further incubated at 37°C for an additional 24 h. Biofilm biomass was qualified using a previously established crystal violet assay, and the remaining biofilm ratio was determined accordingly. Phage cocktail synergy was assessed using the above-described method, with biofilm biomass quantified to determine fractional inhibition.

### Statistical analysis

All graphs were generated using GraphPad Prism version 10, and statistical analyses were performed using the same software. Group differences were assessed using ANOVA, followed by Tukey’s HSD test for multiple comparisons.

## Data Availability

The genome sequence of phages is available in GenBank under accession numbers including: 1. PX668376.1 (phiAR002: https://www.ncbi.nlm.nih.gov/nuccore/PX668376) 2. PX668377.1 (phiAR010: https://www.ncbi.nlm.nih.gov/nuccore/PX668377) 3. PX668378.1 (phiAR014: https://www.ncbi.nlm.nih.gov/nuccore/PX668378).

## SUPPLEMENTAL MATERIAL

Supplemental material spectrum.04009-25-s0001.pdf Fig. S1; Tables S1 to S4.

ASM does not own the copyrights to Supplemental Material that may be linked to, or accessed through, an article. The authors have granted ASM a non-exclusive, world-wide license to publish the Supplemental Material files. Please contact the corresponding author directly for reuse.
