# Supplementary material for: Synergistic effects of halo-plaque forming phage cocktails against polymicrobial Acinetobacter baumannii and Staphylococcus aureus
Source: Microbiol Spectr. 2026 Apr 30;14(6):e04009-25. doi: 10.1128/spectrum.04009-25 (PMC13227992; doi:10.1128/spectrum.04009-25)
Supplement: Supplemental material — Fig. S1; Tables S1 to S4. [file spectrum.04009-25-s0001.pdf]

A

Bacteria	Strain code	phiAR002	phiAR004	phiAR010	phiAR011	phiAR014	phiAR015
Parental host	PT061	■	■	■	■	■	■
Acinetobacter baumannii	PT007	■	■		■		
	PT027			■			
	PT049			■	■		
	PT054	■	■	■	■		
	PT058	■	■	■	■		
	PT046			■	■		
	PT003	■	■	■	■	■	■
	PT009	■					
	PT022		■	■	■		
	PT039	■		■	■		
	PT020	■			■		
	PT006					■	■
	PT018					■	
	PT028					■	■
	PT035					■	■
	PT040	■	■	■	■	■	■
	PT052	■	■	■	■		■
	PT062	■				■	■
	PT064						■
	PT071					■	■
	PT073					■	■
	PSU091			■			
	PSU075			■	■		
	PSU079						
	PSU084	■	■		■		
	PSU051		■	■	■		
	PSU052	■	■	■	■		
	PSU068			■	■	■	■

B

Bacteria	Strain code	phiAR002	phiAR004	phiAR010	phiAR011	phiAR014	phiAR015
Acinetobacter baumannii	PSU128			■			
	PSU048						■
	PSU054			■	■		
	PSU081	■					
	PSU083	■					
	PSU088	■					■
	PSU131	■	■	■	■		
	PSU078	■		■	■		
	PSU127						■
	PSU087						■
	PSU125	■				■	■
	PSU137					■	■
	PSU139	■					■
	PSU104	■	■	■	■		
	PSU122				■		■
	PSU133	■		■	■		
	PSU142				■		
	DMST 2071	■	■				
	ATCC19606	■	■	■	■		
Staphylococcus aureus	AR009						
	AR010						
	AR011						
	AR012						
	AR013						
	AR014						
	ATCC29312						
Pseudomonas aeruginosa	ATCC 27853						
Klebsiella pneumoniae	ATCC700603						
Escherichia coli	ATCC25922						

C

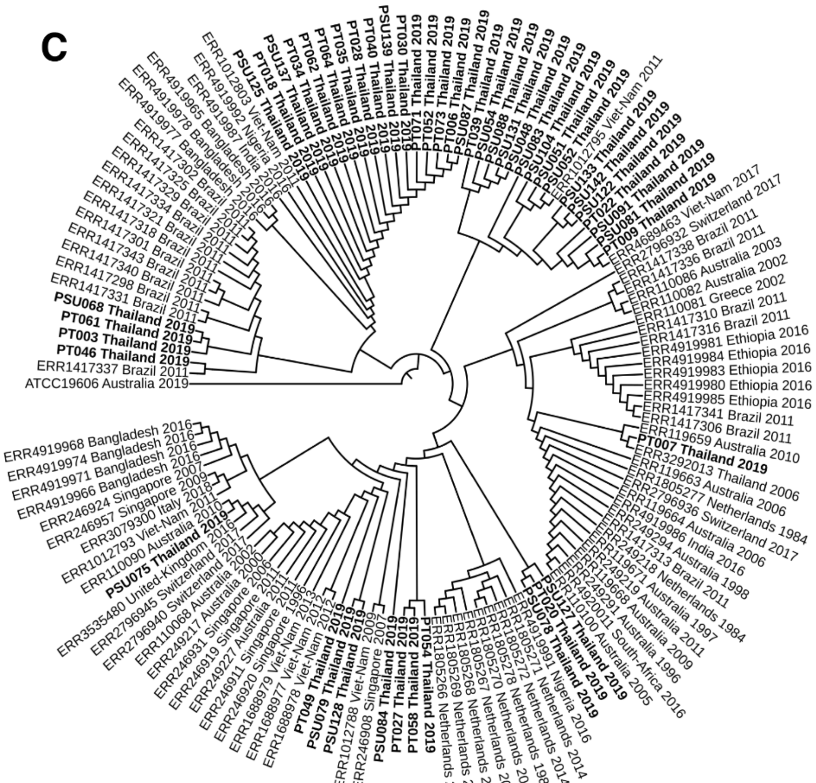

Figure S1. An analysis of host range and phylogenetic relationships of clinical CRAB (carbapenem-resistant *Acinetobacter baumannii*) isolates was conducted through comparison with reference databases

and among the isolates themselves. (**A**) The host range of the isolated bacteriophages was assessed. (**B**) Phylogenetic relationships among CRAB isolates were determined using multilocus sequence typing (MLST) based on the Oxford scheme.

Table S1 List of annotated proteins from ORFs of phiAR002

	CDS	Start	Stop	Direction	E-value	Per. Identities	Sequence similarity	Predicted function
1	1	226	1884	+	2.00E-27	34.96%	XRA13090.1	MAG: Single strand DNA binding protein
2	2	2028	3545	+	4.00E-84	38.11%	XRA13091.1	UvsX-like recombinase
3	4	3843	4997	+	3.00E-23	29.35%	QJT70992.1	Metallophos_2 domain-containing protein
4	7	5779	7026	+	2.00E-36	34.17%	YP_010303000.1	clamp loader subunit, DNA polymerase accessory protein
5	9	7499	9742	+	5.00E-21	23.08%	YP_006382649.1	putative ATPase involved in DNA repair
6	12	11096	12283	+	5.00E-55	30.86%	YP_009290869.1	thymidylate synthase
7	14	13918	14670	+	2.00E-07	44.78%	CAB4222183.1	FolA Dihydrofolate reductase
8	27	24129	24464	+	4.00E-03	32.84%	MEE0596780.1	helix-turn-helix transcriptional regulator
9	31	26380	27087	-	4.00E-78	63.79%	WP_032064940.1	dCTP deaminase
10	33	28414	28959	-	4.00E-36	39.29%	YP_010347965.1	tail assembly chaperone
11	34	29034	31973	-	2.00E-09	40.98%	YP_004009532.1	tail collar fiber protein
12	35	31988	34972	-	3.00E-07	30.86%	XOS25853.1	MAG: virion structural protein
13	36	35050	40125	-	1.00E-23	31.58%	XOS25853.1	MAG: virion structural protein
14	38	40933	42798	-	1.00E-40	26.86%	DAY69060.1	MAG TPA: baseplate wedge protein
15	46	46643	47593	+	5.00E-30	28.85%	SBV38272.1	DNA polymerase I
16	69	61384	62085	+	8.00E-32	38.81%	XTJ53630.1	deoxynucleoside monophosphate kinase
17	80	72573	73247	-	1.20E-02	28.99%	YP_006382584.1	tail sheath
18	81	73257	74600	-	1.00E-20	32.75%	YP_009904638.1	PmgG-like head morphogenesis
19	82	74615	77347	-	3.00E-22	26.85%	DAP09405.1	MAG TPA: lysozyme
20	83	77625	78563	-	2.00E-09	31.68%	XOR59565.1	MAG: baseplate hub assembly catalyst
21	84	78584	79411	-	7.00E-03	27.10%	QRE00346.1	tail fiber protein
22	88	85287	85808	-	5.00E-24	31.18%	XOR58489.1	MAG: baseplate tail tube initiator
23	94	90466	91560	-	9.00E-97	40.72%	QJT71112.1	putative major capsid protein
24	95	91659	92324	-	6.00E-20	31.20%	YP_001950010.1	virion structural protein
25	96	92390	94027	-	3.40E-02	23.57%	WYW02749.1	ATPase
26	99	96180	96593	-	1.00E-25	35.66%	DAL50201.1	MAG TPA_asm: HNH endonuclease
27	101	97967	99508	-	3.00E-75	33.89%	QJT71081.1	DNA topoisomerase IV subunit A
28	105	101539	103464	-	3.00E-125	38.45%	XOR60427.1	MAG: topoisomerase II large subunit
29	109	104754	105020	-	1.00E-33	59.09%	YP_009886405.1	DksA-like zinc-finger protein
30	127	116995	118158	-	1.00E-121	50.00%	QJT70911.1	lysine 2,3-aminomutase
31	130	120459	121235	-	4.00E-10	28.12%	XYN88958.1	MAG TPA: Queuosine biosynthesis protein QueC
32	131	121228	121908	-	4.00E-17	30.10%	YP_009903613.1	QueC-like queuosine biosynthesis
33	133	122319	124244	-	3.00E-35	50.00%	DAN02899.1	MAG TPA: Type I restriction enzyme
34	134	124251	124820	-	1.00E-37	44.19%	YP_009125177.1	baseplate hub subunit and tail lysozyme
35	137	125717	127648	-	1.00E-31	26.57%	XOS26001.1	ribonucleotide reductase small subunit

Table S1 List of annotated proteins from ORFs of phiAR002 cont.

	CDS	Start	Stop	Direction	E-value	Per. Identities	Sequence similarity	Predicted function
36	138	127662	128279	-	2.00E-24	44.64%	DAI28141.1	MAG TPA: zinc ribbon domain protein
37	142	129453	131423	-	2.00E-66	30.43%	QJT71059.1	DNA ligase
38	151	138852	139457	-	8.00E-19	29.29%	YP_009904178.1	Holliday junction resolvase
39	152	139441	140634	-	2.00E-37	29.46%	QJT70838.1	6-pyruvoyl tetrahydropterin synthase-like protein
40	155	143419	145518	+	2.00E-20	25.78%	YP_006382733.1	putative topoisomerasae I protein
41	157	146364	147515	+	1.00E-26	34.62%	YP_001949993.1	head-tail connector protein
42	163	150631	150966	+	8.00E-12	40.23%	WP_262020261.1	HNH endonuclease
43	165	151422	156917	+	1.00E-106	30.10%	WYW01052.1	DNA helicase
44	168	158171	161272	+	3.00E-112	29.70%	YP_009903599.1	exonuclease
45	169	161332	162351	+	5.00E-13	32.17%	DAV58077.1	putative 7-cyano-7-deazaguanosine (preQ0) biosynthesis protein QueE
46	173	164927	165619	+	3.00E-24	34.48%	DAH47853.1	MAG TPA: GTP cyclohydrolase I
47	176	166302	168029	+	2.00E-56	30.44%	YP_009903596.1	ATP-dependent DNA helicase
48	177	168026	168628	+	5.00E-03	26.76%	WOL24399.1	DNA polymerase
49	178	168625	170046	+	8.00E-90	34.26%	WYW00917.1	DnaB-like replicative helicase
50	180	170641	172899	+	1.00E-125	34.39%	WYW01395.1	terminase large subunit
51	182	173226	175016	+	2.00E-110	37.14%	YP_008433438.2	portal protein
52	183	175112	176035	+	1.00E-55	44.33%	SBV38416.1	Phage endolysin
53	188	179941	180270	-	2.00E-24	47.25%	XTJ53740.1	membrane protein
54	189	180334	181341	-	6.00E-38	29.28%	QRE00077.1	DNA primase
55	192	182462	183469	+	2.00E-20	28.10%	DAY69083.1	MAG TPA: PD-(D/E)XK nuclease superfamily protein
56	193	183523	184542	+	2.00E-17	23.26%	YP_008433505.2	peptidase
57	194	184600	185310	+	1.00E-13	27.68%	YP_010675207.1	L-glutamine-D-fructose-6-phosphate aminotransferase
58	195	185312	186928	+	4.00E-20	26.67%	YP_009903615.1	tRNA guanine transglycosylase

Table S2 List of annotated proteins from ORFs of phiAR010

	CDS	Start	Stop	Direction	E-value	Per. Identities	Sequence similarity	Predicted function
1	1	202	1728	+	-5.03E+09	98.23%	UNI74600.1	short tail fiber
2	2	1728	3593	+	-5.48E+16	99.52%	WBF78481.1	SGNH hydrolases
3	3	3625	4542	+	4.00E-180	91.15%	CAL4858321.1	head-tail adaptor Ad2
4	4	4546	5310		3.00E-179	93.31%	UQS94246.1	neck protein
5	5	5389	6165	+	1.00E-161	99.60%	YP_009886444.1	tail terminator
6	6	6165	6647	+	5.00E-111	93.75%	YP_009881530.1	terminase small subunit
7	7	6644	7081	+	5.00E-98	94.48%	YP_009886696.1	putative DNA packaging protein
8	8	7032	8888	+	-1.40E+13	97.61%	QQO96372.	terminase large subunit
9	9	8919	10895	+	-9.42E+15	99.70%	YP_009885357.1	tail sheath
10	10	10932	11423	+	8.00E-120	98.77%	YP_009889807.1	tail protein
11	11	11484	13037	+	-5.43E+13	99.42%	YP_009880946.1	portal protein
12	12	13037	13240	+	3.00E-38	100.00%	YP_009881524.1	prohead
13	13	13240	13668	+	1.00E-98	99.30%	YP_009880948.1	head scaffolding protein
14	14	13665	14312	+	5.00E-156	99.53%	YP_009881522.1	head maturation protease
15	15	14342	15175	+	7.00E-152	99.28%	UQS93762.1	prohead core protein
16	16	15205	16785	+	-4.67E+14	99.43%	WBF78490.1	major capsid protein
17	17	16818	18248	-	-1.45E+11	98.94%	UQS94011.1	endonuclease
18	18	18330	19601	+	-3.78E+11	97.64%	CAL1777118.1	major head protein
19	19	19664	20749	-	-1.21E+07	100.00%	UJH94973.1	putative outer capsid protein
20	20	20759	21457	-	1.00E-167	99.14%	WBF78540.1	inhibitor of prohead protease protein
21	21	21539	23050	+	-1.69E+12	100.00%	YP_009881515.1	DNA helicase
22	27	24526	24924	-	1.00E-93	97.73%	UNI74628.1	baseplate wedge subunit
23	28	24924	25568	-	6.00E-156	99.07%	YP_009880963.1	baseplate hub
24	29	25636	26391	+	-2.43E+06	98.80%	WYA89040.1	baseplate hub
25	30	26388	27971	+	-2.24E+14	98.80%	WPH64874.1	baseplate hub
26	31	27973	28521	+	3.00E-133	99.45%	YP_009885380.1	baseplate hub distal subunit
27	32	28514	30238	+	-8.08E+12	99.65%	YP_009880967.1	baseplate hub subunit and tail length
28	33	30248	31339	+	-4.35E+07	99.45%	YP_009886970.1	baseplate tail tube cap
29	34	31336	32214	+	-1.94E+07	98.97%	WYA89045.1	tail tube
30	35	32238	34319	+	-2.92E+18	99.28%	QKE55901.1	RNA polymerase ADP-ribosylase
31	36	34537	36027	-	-1.22E+15	99.60%	YP_009885386.1	DNA ligase
32	42	37840	38082	-	5.00E-49	100.00%	YP_009880976.1	lysis inhibition; accessory protein
33	43	38159	38488	-	7.00E-68	93.27%	YP_009882204.1	head morphogenesis
34	45	38826	39377	-	6.00E-135	99.45%	YP_009886734.1	dCMP deaminase
35	46	39739	40266	-	6.00E-124	94.89%	YP_009887139.1	MutT/NUDIX hydrolase

Table S2 List of annotated proteins from ORFs of phiAR010 cont.

	CDS	Start	Stop	Direction	E-value	Per. Identities	Sequence similarity	Predicted function
36	48	40266	41186	-	-1.03E+07	96.41%	QGT54223.1	polynucleotide 5'-kinase and 3'-phosphatase
37	51	42068	42604	-	2.00E-118	100.00%	WPH64896.1	inhibitor of host transcription
38	54	42855	44000	-	-1.24E+08	98.69%	QGT54229.1	RNA ligase A
39	55	44308	45459	-	-4.30E+08	99.48%	QGT54230.1	aerobic NDP reductase small subunit
40	56	45519	47930	-	-3.63E+18	99.75%	UQS94050.1	aerobic ribonucleoside diphosphate reductase, large subunit
41	59	48434	49381	-	-8.17E+08	99.68%	UYL85996.1	thymidylate synthase
42	61	49643	50194	-	2.00E-135	98.91%	WBF78804.1	dihydrofolate reductase protein
43	62	50196	50474	-	1.00E-62	98.91%	YP_009886500.1	inhibitor of host Lon protease
44	66	51481	52407	-	-1.28E+06	99.35%	YP_009886504.1	single strand DNA binding protein
45	67	52537	53187	-	5.00E-156	98.61%	WPH64912.1	DNA helicase loader
46	68	53180	53479	-	7.00E-54	80.81%	CAL1777316.1	RNA polymerase-associated protein
47	69	53460	53726	-	4.00E-46	95.45%	YP_009882178.1	transcriptional regulator
48	70	54023	55135	-	-1.64E+10	99.72%	CAL1778056.1	exonuclease
49	72	55340	59260	+	-1.13E+24	98.55%	YP_009885421.1	tail fiber protein proximal subunit
50	73	59270	60400	+	-2.11E+07	98.40%	QKE55938.1	hinge connector of long tail fiber, proximal connector
51	74	60456	61169	+	1.00E-171	99.58%	CAL4858241.1	hinge connector of long tail fiber protein distal connector
52	75	61182	64922	+	-1.38E+15	91.98%	CAH1067986.1	tail fiber protein
53	76	65217	65948	+	8.00E-180	97.53%	UNI74677.1	holin
54	77	65949	66224	-	2.00E-58	95.60%	QKE55943.1	anti-sigma 70 protein
55	78	66236	66853	-	1.00E-147	98.05%	CAL1778029.1	MotA-like activator of middle period transcription
56	79	66941	68284	-	-5.52E+11	99.55%	YP_009886273.1	DNA topoisomerase II
57	81	68535	68993	-	2.00E-110	98.68%	YP_009885431.1	Ndd-like nucleoid disruption protein
58	82	69056	69658	-	5.00E-149	99.00%	YP_009882164.1	DenB-like DNA endonuclease IV
59	85	71148	73355	-	-9.81E+17	98.23%	WBF78725.1	RIIA lysis inhibitor
60	89	74022	75290	-	-2.00E+11	94.31%	YP_006488997.1	glutathionylspermidine synthase
61	92	76313	76654	-	2.00E-72	98.23%	YP_009885191.1	quaternary ammonium compound-resistance protein QacE
62	93	76809	77816	-	-4.25E+09	99.40%	CAL4858221.1	RNA ligase
63	96	78415	80232	-	-3.77E+15	98.84%	XOT52495.1	DNA topoisomerase large subunit
64	97	80311	80877	-	9.00E-136	98.40%	UQS93845.1	endolysin
65	99	81332	81574	-	3.00E-51	98.75%	WPH64950.1	modifier of supressor tRNAs
66	100	81670	82329	-	1.00E-162	98.63%	YP_009881430.1	exonuclease A
67	105	83393	84751	-	-6.06E+09	98.45%	YP_009880794.1	Dda-like helicase
68	109	85886	86839	-	-2.76E+07	99.05%	YP_009886302.1	Srd anti-sigma factor
69	111	87240	87683	-	4.00E-107	99.32%	WYA88865.1	phosphatase
70	120	89953	90426	-	2.00E-83	85%	WUV29483.1	DNA binding protein

Table S2 List of annotated proteins from ORFs of phiAR010 cont.

	CDS	Start	Stop	Direction	E-value	Per. Identities	Sequence similarity	Predicted function
71	127	92941	93450	-	4.00E-124	98.22%	YP_009886816.1	DprA-like DNA recombination-mediator protein
72	131	94538	95056	-	1.00E-125	98.84%	YP_009880819.1	nucleoside triphosphate pyrophosphohydrolase
73	140	98710	99738	-	-1.91E+07	92.98%	WPH64995.1	DNA primase
74	141	99735	100406	-	5.00E-166	99.55%	WBF78790.1	GIY-YIG endonuclease
75	155	105454	106950	-	-4.25E+14	99.80%	YP_009885253.1	replication and recombination DNA helicase
76	156	107019	107321	-	3.00E-65	96.00%	CAL1777749.1	head vertex assembly chaperone
77	159	109622	110164	-	2.00E-127	98.89%	UQS93656.1	thymidylate kinase
78	161	111100	111807	-	4.00E-177	99.57%	YP_009889705.1	thymidylate synthase
79	162	111807	112187	-	1.00E-89	99.20%	YP_009886351.1	phosphoheptose isomerase
80	164	113916	114545	-	2.00E-156	99.52%	YP_009886852.1	arabinose 5-phosphate isomerase
81	171	116737	117909	-	-1.32E+12	99.74%	UNI74768.1	DNA polymerase
82	178	119915	121423	-	-3.01E+09	99.20%	UNI74768.1	DNA polymerase
83	179	121734	122117	-	2.00E-90	99.21%	UQS93924.1	translation repressor protein
84	181	122114	122683	-	1.00E-137	98.94%	UNI74777.1	clamp loader small subunit
85	182	122745	123725	-	-2.93E+09	99.69%	YP_009886126.1	clamp loader of DNA polymerase
86	183	123777	124466	-	6.00E-168	99.56%	YP_009881603.1	DNA polymerase accessory protein, sliding clamp
87	184	124505	124879	-	2.00E-88	99.19%	YP_009886128.1	RNA polymerase binding
88	185	125551	127236	-	-3.09E+10	99.64%	YP_009886876.1	SbcC-like subunit of palindrome specific endonuclease
89	189	127233	127844	-	7.00E-152	99.01%	YP_009880876.1	phosphoesterase
90	190	128098	129108	-	-1.34E+08	99.40%	YP_009885289.1	SbcD-like subunit of palindrome specific endonuclease
91	194	129692	130234	-	1.00E-131	97.78%	XEO41493.1	RNA polymerase sigma factor for late transcription
92	198	131258	131740	-	2.00E-115	99.38%	QGT54124.1	endonuclease VII
93	214	139428	140468	-	-2.48E+06	95.04%	XEO41448.1	putative nucleotidyltransferase
94	215	140812	141273	-	2.00E-63	93.07%	YP_009886653.1	membrane protein
95	217	141279	141545	-	3.00E-61	97.73%	YP_009886405.1	DksA-like zinc-finger protein
96	218	141607	141885	-	8.00E-64	98.91%	YP_009886406.1	lysis inhibition
97	221	142603	142932	-	2.00E-75	100.00%	UQS93964.1	Vs valyl-tRNA synthetase modifier
98	223	143580	144017	-	8.00E-79	99.13%	YP_009886166.1	homing endonuclease
99	233	147262	147828	-	4.00E-131	95.74%	YP_009881557.1	tail fiber protein
100	234	147905	148141	-	6.00E-52	98.72%	UNI74580.1	peptide chain release factor
101	242	149570	150025	-	2.00E-76	69.80%	WJJ54793.1	anti-cbass nuclease
102	243	150025	150264	-	6.00E-49	100.00%	YP_009880921.1	tail fiber chaperone
103	244	150251	150988	-	6.00E-173	97.48%	YP_009886922.1	deoxynucleoside monophosphate kinase
104	245	150985	151554	-	3.00E-139	99.47%	YP_009886923.1	tail completion protein
105	246	151783	152622	-	-5.58E+07	98.92%	UNI74588.1	DNA end protector

Table S2 List of annotated proteins from ORFs of phiAR010 cont.

	CDS	Start	Stop	Direction	E-value	Per. Identities	Sequence similarity	Predicted function
106	247	152631	153086	-	6.00E-107	96.69%	CAL1777293.1	head closure
107	248	153128	153733	+	4.00E-145	99.49%	YP_009886429.1	baseplate wedge subunit
108	249	153714	155498	+	-1.08E+15	99.33%	YP_009886430.1	baseplate hub subunit and tail lysozyme
109	251	155962	156237	+	9.00E-62	100.00%	YP_009880929.1	PAAR motif of membran proteins
110	252	156247	158196	+	-8.32E+16	94.30%	CAL1777716.1	baseplate wedge subunit
111	253	158193	161285	+	-2.73E+20	99.22%	UNI74841.1	baseplate wedge subunit
112	254	161278	162282	+	-1.51E+07	89.67%	WJJ54805.1	baseplate wedge subunit
113	255	162342	163208	+	-5.13E+06	99.31%	CAL1777245.1	baseplate wedge tail fiber protein connector
114	256	163208	165025	+	-7.31E+14	99.67%	YP_009881537.1	baseplate wedge subunit

Table S3 List of annotated proteins from tRNA of phiAR010

		Start	Stop	tRNA	E-value
1	pseudogene	145754	145825	Trp(CCA)	45.5
2	tRNA	146212	146284	Phe(GAA)	39.9
3	tRNA	146297	146370	Pro(TGG)	62.2
4	tRNA	146695	146768	Asn(GTT)	61.4
5	pseudogene	146875	146942	Thr(TGT)	18.3
6	pseudogene	148506	148580	Leu(TAG)	36.3
7	tRNA	148660	148735	Cys(GCA)	49
8	tRNA	148746	148817	Met(CAT)	48.3
9	tRNA	148830	148901	Sup(CTA)	62.3

Table S4 List of annotated proteins from ORFs of phiAR014

	CDS	Start	Stop	Direction	E-value	Per. Identities	Sequence similarity	Predicted function
1	17	19312	20505	+	2.00E-21	39.02%	XOS33723.1	peptidase
2	20	21751	22896	+	3.00E-06	31.18%	YP_010670967.1	MutT/NUDIX hydrolase
3	25	28317	29279	-	1.00E-38	41.35%	XOR75159.1	head maturation protease
4	26	29429	30331	-	8.00E-17	33.51%	YP_009103660.1	head maturation protease
5	35	41880	43184	-	7.00E-12	21.31%	DAJ86512.1	MAG TPA: Glutamate decarboxylase
6	53	65982	69356	-	1.00E-131	30.56%	AUR91490.1	P-loop containing nucleoside triphosphate hydrolase
7	69	85793	86347	-	4.00E-106	79.89%	YP_009055463.1	endolysin
8	73	88080	90374	-	5.8267E+14	50.42%	QOV07848.1	tailspike protein
9	75	92653	93270	-	2.00E-62	67.57%	XOS00398.1	putative phage lysozyme
10	77	94627	95265	-	4.00E-37	44.44%	YP_009222579.1	DNA ejection
11	78	95262	95708	-	1.00E-05	29.37%	CAB4139390.1	acetyltransferase
12	81	96425	97819	-	5.00E-96	36.11%	XOF02173.1	MAG: head closure Hc3
13	82	97827	98438	-	4.00E-09	24.42%	UOF78617.1	portal protein
14	84	98917	100149	-	5.00E-110	44.58%	XOS00666.1	major capsid protein
15	86	100925	103174	-	1.00E-113	33.42%	DAP51275.1	portal protein
16	87	103165	104616	-	7.4318E+10	99.33%	WP_322853059.1	phage terminase large subunit family protein
17	88	104594	105055	-	7.00E-88	81.70%	WP_347016444.1	terminase small subunit
18	90	105216	105698	-	6.00E-25	40.30%	WBF05275.1	NinB/ Orf homologous recombination mediator
